# TPPU protects against seizures and seizure-associated comorbidities by inhibiting the Akt/mTOR signaling pathway in KA-induced convulsant mice

**DOI:** 10.3389/fimmu.2026.1850303

**Published:** 2026-06-08

**Authors:** Rong Tang, Banglian Hu, Sulan Xie, Yijun Shen, Ziwei Wang, Xingyi Wang, Xiaohua Huang, Guoqiang Fei, Weifeng Peng, Honghua Zheng, Jing Ding, Xin Wang

**Affiliations:** 1 Department of Neurology, Zhongshan Hospital (Xiamen), Fudan University, Xiamen, China; 2 Department of Neurology, Zhongshan Hospital, Fudan University, Shanghai, China; 3 Fujian Provincial Key Laboratory of Neurodegenerative Disease and Aging Research, Institute of Neuroscience, School of Medicine, Xiamen University, Xiamen, China; 4 Department of Neurology, Shanghai Geriatric Medical Center, Shanghai, China; 5 Basic Medical Sciences, School of Medicine, Xiamen University, Xiamen, Fujian, China

**Keywords:** Akt/mTOR axis, astrocytes, comorbidities, epilepsy, neuroinflammation, TPPU

## Abstract

**Background:**

Shared pathophysiological mechanisms exist between epilepsy and its associated comorbidities, with neuroinflammation playing a key role. 1-Trifluoromethoxyphenyl-3-(1-propionylpiperidin-4-yl) urea (TPPU) is a soluble epoxide hydrolase (sEH) inhibitor that exhibits potent, broad-spectrum anti-inflammatory effects by preventing the hydrolysis of epoxyeicosatrienoic acids (EETs). However, the potential anti-epileptic and comorbidity-alleviating effects of TPPU, along with the underlying molecular mechanisms, remain to be elucidated.

**Methods:**

Electroencephalogram (EEG) recordings and Racine score were used to monitor seizures. Behavioral tests were employed to assess seizure-associated cognitive and anxiety-like comorbidities in mice. Whole-cell patch-clamp recordings were used to evaluate synaptic function. RNA-sequencing was conducted to elucidate the molecular mechanisms underlying the neuroprotective effects of TPPU in the KA-induced chronic epileptic model.

**Results:**

Behavioral assessments, EEG monitoring, and whole-cell patch-clamp recordings revealed that TPPU significantly mitigated seizure severity and anxiety/depressive-like behaviors, and enhanced cognitive function in kainic acid (KA)-induced chronic epileptic mice. TPPU exhibited neuroprotective properties by reducing neuronal apoptosis and neuroinflammation. Bulk RNA-sequencing analysis indicated that TPPU protected against neuroinflammation during epileptogenesis. Mechanistic investigations revealed that TPPU suppresses Akt/mTOR pathway activation.

**Conclusions:**

Our findings establish the Akt/mTOR axis as a critical pathway mediating the protective actions of TPPU against epilepsy and its comorbidities in murine models. This work not only advances our understanding of epilepsy pathophysiology but also identifies novel targets for the development of comprehensive therapeutic strategies.

## Introduction

Epilepsy is a chronic neurological disorder characterized by recurrent central nervous system (CNS) dysfunction caused by abnormally synchronized neuronal discharges (1). As one of the most common neurological diseases, epilepsy affects around 50 million people globally, according to WHO estimates (2). The disorder has an annual incidence of 50–60 cases per 100,000 population (3), with approximately 5 million new cases emerging worldwide each year (WHO, 2024) (4). Beyond seizure manifestations, patients with epilepsy frequently experience neuropsychiatric comorbidities including anxiety, depression, cognitive dysfunction, and migraines (5). Neuroinflammation has emerged as a key contributor to the shared pathophysiological mechanisms between epilepsy and these comorbidities, suggesting that targeting neuroinflammatory processes may represent a promising therapeutic strategy for developing disease-modifying treatments for epilepsy (6).

Recent research demonstrates that pharmacological inhibition (via small molecules) or genetic ablation (through knockout techniques) can effectively suppress soluble epoxide hydrolase (sEH) activity, leading to the stabilization and increased levels of epoxyeicosatrienoic acids (EETs) and other cardioprotective epoxy fatty acids (EpFAs) (7–9). 1-Trifluoromethoxyphenyl-3-(1-propionylpiperidin-4-yl)urea (TPPU) is a potent sEH inhibitor that blocks the degradation of EETs, thereby prolonging their biological half-life and exerting anti-inflammatory activity (10, 11).

Growing evidence indicates the neuroprotective potential of TPPU in Alzheimer’s disease, anxiety/depression, and neuropathic pain (12–15). Our previous findings demonstrate that TPPU attenuates spontaneous seizures, depressive behaviors and inflammation in a lithium-pilocarpine rat model (16, 17). However, the precise molecular mechanisms underlying the antiepileptic activity of TPPU and the associated neuropsychiatric comorbidities, including anxiety, depression, and cognitive dysfunction, require further elucidation.

## Materials and methods

### Reagents

TPPU and kainic acid were bought from Med Chem Express (MCE). Anti sEH was from Santa Cruz Biotechnology. Anti-GAPDH (RRID: AB_2630358) was from Abcam. Anti-CSF1R (RRID: AB_884158) was from R&D Systems. Iba1/AIF-1(RRID: AB_2493179) was purchased from Synaptic systems. Anti-GFAP (RRID: AB_2109646) was from Proteintech. Anti-CD68 was purchased from Biolegend. Phospho-mTOR (Ser2448) (D9C2) XP^®^ Rabbit mAb, mTOR (7C10) Rabbit mAb, Phospho-Akt (Ser473) (D9E) XP^®^ Rabbit mAb, and Akt Antibody were bought from Cell Signaling Technology (CST). Donkey anti-Rabbit IgG (H+L) Highly Cross-Adsorbed Secondary Antibody, Alexa Fluor™ 488, donkey anti-mouse IgG (H+L) Highly Cross-Adsorbed Secondary Antibody, Alexa Fluor™ 568 and goat anti-Guinea Pig IgG (H+L) Highly Cross-Adsorbed Secondary Antibody, Alexa Fluor™ 647 were from Invitrogen. Poly-L-lysine (PLL, P1274) and TritonTM X-100 (T8787) were purchased from Sigma. 100×proteinase inhibitor cocktail (k1007) and 100×phosphatase inhibitor cocktail (k1015) were all from APE×BIO. RNA isolater Total RNA Extraction Reagent (R401-01), HIScript III All-in-one RT SuperMix Perfect for qPCR (R333-01), HamQ Universal SYBR qPCR Master Mix (q711-03) and TUNEL assay kit were all from Vazyme. DMEM (PM150210) was bought from Pricella. 1% penicillin/streptomycin (S110JV) was from BasalMedia. Fetal bovine serum (FBS, FSD500) was from ExCell Bio. RIPA buffer (R1091) was bought from LABLEAD. Protein Quantification Kit (BCA Assay) and SuperKine ECL detection kit (BMU102-CN) were from Abbkine.

### Epilepsy model and seizure monitoring

**Figure 1:**
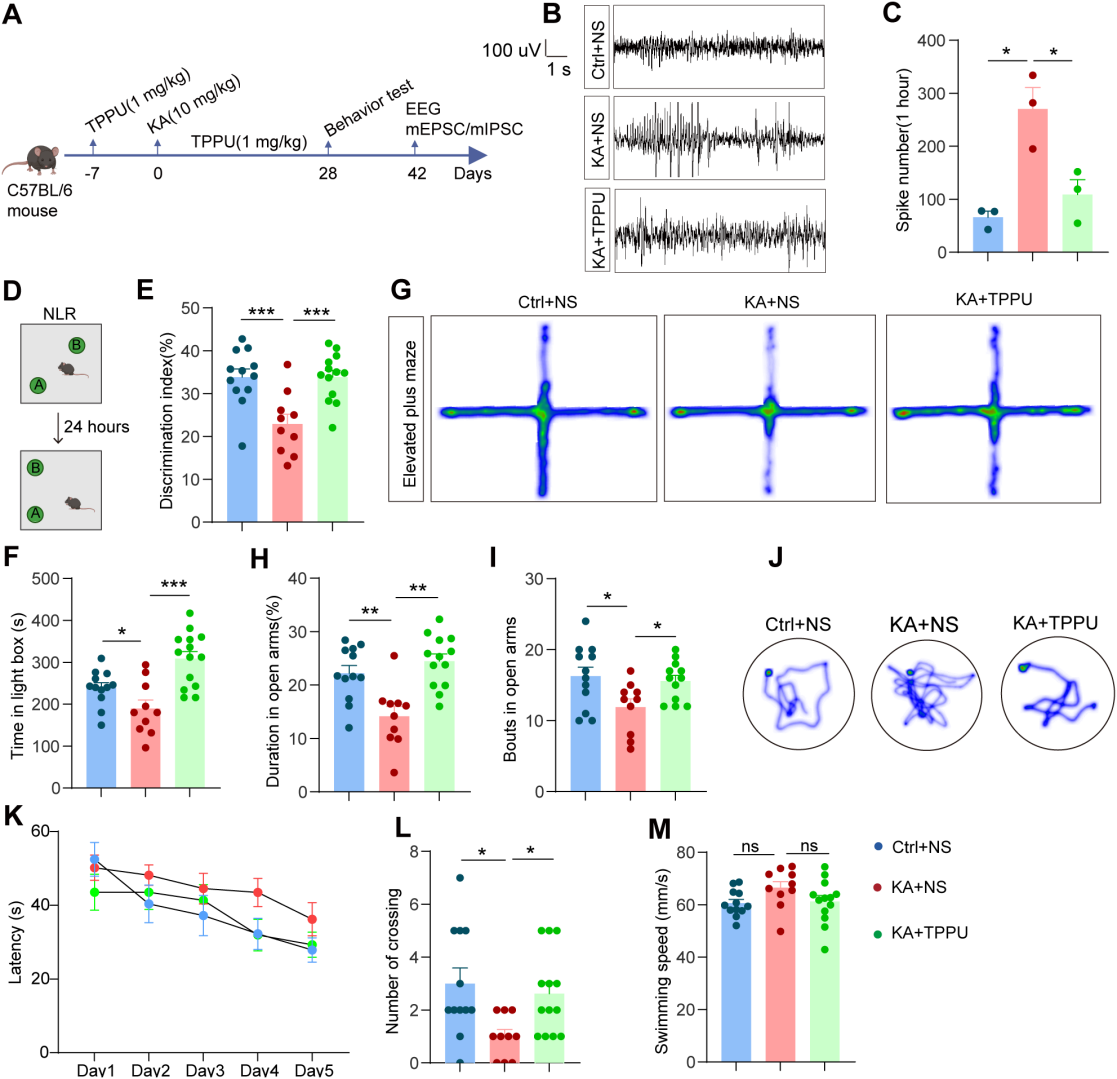
TPPU treatment significantly ameliorates both seizure severity and the related comorbidities in chronic epileptic mouse models. **(A)** Schematic time line of TPPU administration in KA-induced chronic epileptic mouse model. **(B)** Representative EEG recordings from different groups. **(C)** Statistical analysis of epileptiform discharge frequency (spike number) in different groups. **(D, E)** Novel location recognition experiment: On the first day, the time spent by the mice exploring two objects, A and B, was recorded. On the second day, object B was moved to a new location, same distance away from the walls. The number of times and the time spent exploring objects A and B within 10 minutes were recorded and analyzed. The novel location recognition index for the three groups of mice was calculated as Discrimination index = Novel location B/(A + Novel location B) × 100%, n=10-13. ***p < 0.001. **(F)** Black and white box test: The duration spent in the white box within 10 minutes was recorded for the three groups of mice, n=10-13. **(G–I)** Elevated plus maze test: The duration **(H)** and number **(I)** of entries into the open arms within 10 minutes were recorded in those mice, n=10-13. **(J, K)** Morris water maze experiment: From days 1 to 5, a training phase was conducted, recording the time taken by the control group, epileptic group, and TPPU treatment group mice to find the hidden platform within 1 minute, n=10-13. **(L, M)** Morris water maze experiment: On day 6, a testing phase was conducted where the platform was removed, and the number of times the mice from the control group, epileptic group, and TPPU treatment group crossed the platform and the time spent in the target quadrant within 1 minute were recorded, n=10-13. *p < 0.05. One-way ANOVA, ns: no statistical difference; *p<0.05, **p<0.01, ***p<0.001.

Eight-week old adult male C57BL/6 mice were randomly assigned to three groups: control+saline (Ctrl+NS), kainic acid (KA) +saline (KA+NS), and KA+TPPU (KA+TPPU). Two distinct treatment schedules are explicitly defined. For the protocol of acute epilepsy model (experimental scheme diagram, **Supplementary Figure 1A**), mice in the KA+TPPU group were pre-treated with 1 mg/kg/d TPPU (100 μL) (dissolved by sonication in a vehicle of 10% DMSO, 40% PEG400, and 50% saline according to the instructions, intraperitoneal injection) for 3 consecutive days, whereas the Ctrl+NS and KA+NS groups received an equivalent volume of saline. For the protocol of chronic epilepsy model (experimental scheme diagram, **Figure 1A**), mice in the KA+TPPU group were pre-treated with 1 mg/kg/d TPPU for 7 consecutive days, while the Ctrl+NS and KA+NS groups received an equivalent volume of saline. On day 4 (acute model) or day 8 (chronic model), the KA+NS and KA+TPPU groups were administered 10 mg/kg kainic acid (KA) via intraperitoneal injection (i.p.), whereas the Ctrl+NS group received the same volume of saline. Approximately 30 min after injection, mice exhibited epileptic seizures. Seizure severity was then assessed according to the Racine scale (18): stage 1, immobility and facial twitching; stage 2, head nodding; stage 3, forelimb clonus; stage 4, rearing; and stage 5, rearing and falling. Mice that did not reach stage 4 seizures within 1h were given an additional dose of 5 mg/kg KA. Animals that still failed to exhibit stage 4–5 seizures after two supplemental injections were excluded from the study. To reduce mortality, mice sustaining stage 4–5 seizures for more than 30 min were administered 10 mg/kg diazepam (i.p.) to terminate seizure activity.

### Drug administration

After epilepsy model establishment, in the acute epilepsy model, the KA+TPPU group continued to receive TPPU (1 mg/kg/d) for an additional 7 days. In the chronic epilepsy model, the KA+TPPU group received TPPU (1 mg/kg/d) for an additional 28 days, while the KA+NS and Ctrl+NS groups were maintained with an equivalent volume of saline. Behavioral tests and electroencephalography (EEG) recordings were conducted thereafter.

### Electroencephalography recording

Electroencephalographic (EEG) recordings were performed on day 8 (acute epilepsy model) or day 29 (chronic epilepsy model) after model establishment and drug administration. Mice were anesthetized with 1.25% tribromoethanol via intraperitoneal injection and secured in a stereotaxic frame. After scalp incision and exposure of the skull, the bregma and lambda were identified. The electrode board was implanted between these landmarks. Four burr holes were drilled at positions matching the fixation holes of the board, and the board was affixed with screws. Bilateral polyamide-insulated stainless steel monopolar microelectrodes (0.1 mm in diameter) were implanted in the frontal cortices of both hemispheres (coordinates: −1.5 mm from bregma, ± 1.8 mm lateral). An additional insulated stainless steel wire (50 μm in diameter) was implanted 1.7 mm below the cortical surface. A reference electrode was placed in the cerebellum. Dental cement was applied around the board for reinforcement. Mice were allowed to recover on a heating pad. Two days after surgery, EEG signals were recorded for 4 hours per mouse using the Pinnacle mouse EEG/EMG recording system. Data were analyzed with Pinnacle Sirenia Seizure software (USA).

### Electrophysiological recording

Mice were anesthetized with isoflurane and decapitated. Brains were rapidly stabilized and sectioned coronally at 400 µm thickness using a vibratome (Leica VT1200S) in ice-cold and perfused with carbogen-saturated artificial cerebrospinal fluid (ACSF) containing: 126 mM NaCl, 18 mM NaHCO_3_, 2.5 mM KCl, 1.2 mM NaH_2_PO_4_, 1.2 mM CaCl_2_, 2.4 mM MgCl_2_, and 11 mM glucose (pH 7.4). Slices were recovered at room temperature (RT) for 30 min, then maintained at room temperature (22-24 °C) for ≥30 min before recording were performed. All solutions were bubbled with 5% CO_2_/95% O_2_. Whole-cell recordings were obtained from cortical neurons. Miniature excitatory postsynaptic currents (mEPSCs) and miniature inhibitory postsynaptic currents (mIPSCs) were recorded at holding potentials of -70 mV and 0 mV, respectively. Recording pipettes were pulled from borosilicate glass capillaries (5–8 MΩ). The extracellular recording solution consisted of 2.5 mM KCl, 126 mM NaCl, 2.4 mM MgCl_2_, 1.2 mM CaCl_2_, 1.2 mM NaH_2_PO_4_, 11 mM glucose, and 18 mM NaHCO_3_ (pH 7.4,-300 mOsm). The pipette was filled with internal solution containing 140 mM CsCH_3_SO_3_, 2 mM MgCl_2_.6H_2_O, 5 mM TEA-Cl, 10 mM HEPES, 1 mM EGTA, 2.5 mM Mg-ATP, and 0.3 mM Na-GTP (pH 7.3,-300 mOsm). Before recording, the perfusion solution was supplemented with 1 mM TTX for mEPSC and mIPSC recordings. For action potential(AP), the pipette was filled with internal solution containing 140 mM K-gluconate, 2 mM MgCl_2_:6H_2_O, 0.1mM CaCl_2_, 10 mM HEPES, 1.1 mM EGTA, 2.5 mM Mg-ATP, and 0.3 mM Na-GTP (pH 7.3,-300 mOsm), All recordings were performed at RT. Data were filtered at 0.5 kHz, sampled at 10 kHz, collected with a Multi-clamp 700B amplifier and analyzed with pClamp 10.6 software (Molecular Devices).

### Primary cells culture

#### Primary astrocyte culture

Cortices from neonatal (P0-P2) mice were dissected, the meninges were removed, and the tissue was dissociated by trypsinization (0.25% trypsin, 37 °C, 15 min). After terminating the dissociation, the cells were plated in poly−D−lysine−coated T75 flasks and cultured in DMEM/F12 supplemented with 10% fetal bovine serum and 1% penicillin-streptomycin. Following 10–14 days, microglia and oligodendrocytes were removed by orbital shaking (200 rpm, 18 h), and the remaining adherent astrocytes were harvested. Astrocytes were then treated with or without KA (100 μM), followed by treatment with or without TPPU (100 μM) for 24 h.

#### Primary microglia culture

Brain tissues from neonatal mice at postnatal days P0–P2 were mechanically dissociated in complete culture medium (DMEM + 10% FBS + 1% penicillin/streptomycin). The brain homogenate was plated into T75 culture flasks pre-coated with poly-L-lysine (PLL) and incubated. After 24 hours, the medium was replaced with complete medium containing 25 ng/ml GM-CSF. Subsequently, 2.5 ml of complete medium supplemented with GM-CSF was added every three days. At day 10, microglia were purified by shaking at 220 rpm for 30 min. Following this method, microglia were harvested every three days. Microglia were collected for the following RT-PCR experiment and Western blot.

#### Primary neuron culture

Embryos were dissected from pregnant mice at embryonic day 14–16 (E14–E16) and decapitated with sterile scissors. The brains were enzymatically dissociated using 0.25% trypsin and mechanically triturated to obtain a single−cell suspension. The dissociated neurons were plated in culture dishes coated with poly−D−lysine. The cultures were maintained in Neurobasal medium supplemented with 2% B27, 1% glutamine, and 1% penicillin−streptomycin. After 7–10 days in culture, the neurons were collected for the following RT-PCR experiment and Western blot.

### Western blot

Brain tissues or primary astrocytes were harvested, and total proteins were extracted using RIPA buffer containing 1×protease and phosphatase inhibitors. Proteins (30 μg for tissues, 20 μg for cells) were loaded into an 8% SDS-PAGE and transferred onto the PVDF membranes (Millipore, IPVH00010), which were sequentially incubated with primary or secondary antibodies. Proteins bands were visualized using Super ECL Detection Reagents and quantified using ImageJ software.

### Immunofluorescent staining

Brain slice samples were fixed with 4% paraformaldehyde (PFA) and were then incubated overnight at 4°C with primary antibodies: mouse anti-Iba1 (1:200) and anti-CD68 (1:200), anti-GFAP (1:500) and anti sHE (1:50). Subsequently, samples were incubated for 1 hour at room temperature with fluorescent secondary antibodies: Alexa Fluor 488-conjugated goat anti-rabbit IgG (1:500), Alexa Fluor 647-conjugated donkey anti-rat IgG (1:500), Alexa Fluor 568-conjugated donkey anti-mouse IgG (1:500). Z-stack confocal images of brain slices were acquired using an Olympus FV1000MPE-B microscope (Japan). Iba1+ microglia were quantified double-blindly using ImageJ software (v2006.02.01), while Iba1+CD68+ double-positive cells per high-power field (HPF) in hippocampal sections were analyzed with Olympus FV10-ASW 4.0 Viewer. Three-dimensional microglial morphology was reconstructed using Imaris software (v9.2.0; Bitplane, UK).

### Quantitative real-time PCR

TRIzol reagent (Vazyme) was used to extract total RNA from mouse brain samples or primary astrocytes. A total of 1 μg RNA was reverse-transcribed into cDNA using ReverTra Ace qPCR RT Master Mix (Vazyme). Quantitative real-time PCR was performed on the LightCycler 480 System (Roche, Mannheim, Germany) using the FastStart Universal SYBR Green Master (Vazyme). The 2–△△Ct was used to calculate relative gene expression after normalization to the Actb internal control. The primer sequences of target genes are included in **Supplementary Table 1**.

### Terminal deoxynucleotidyl transferase mediated dUTP nick end-labeling assay

*In situ* apoptosis was evaluated using a TUNEL assay kit according to the manufacturer’s instructions (Vazyme, A111-02). Briefly, the frozen brains were sliced into 15 μm sections for subsequent experiments. After fixation and permeabilization, the brain slices were incubated with the TUNEL assay mixture and anti-NeuN antibody for 2 h at 37°C. Subsequently, samples were incubated for 1 hr at room temperature with alexa Fluor 568-conjugated donkey anti-mouse IgG (1:500) fluorescent secondary antibodies, and the nuclei were stained with DAPI for 8 min. The TUNEL-labeled neurons were visualized with a fluorescence microscope (Olympus FV1000MPE-B).

### Enzyme linked immunosorbent assay

Mouse cortex or hippocampus was homogenized in RIPA buffer, homogenized tissue samples were centrifuged at 12,000 rpm at 4°C for 20 min, and supernatants were collected for ELISA analysis. 14,15-DHET level was detected according to the manufacturer’s instructions (YUANJU Bio), 14,15-EET level was detected according to the manufacturer’s instructions (Fine Test).

### RNA-sequencing

Transcriptomic RNA libraries were generated from brain tissues of Ctrl+NS, KA+NS, and KA+TPPU mice (N = 5-6), and were sequenced at Novogene (Beijing). RNA sequencing was performed using the PE150 strategy on an Illumina HiSeq 2500/4000 platform, with an average of 10 GB of reads per sample. Differentially expressed genes (DEGs) between brain tissues of Ctrl+NS, KA+NS and KA+TPPU mice were identified and compared using the DESeq2 (v1.30.1) software package (adjusted p-value < 0.05, |log_2_ fold change| > 1). Enrichment analyses, including Gene Ontology (GO), Kyoto Encyclopedia of Genes and Genomes (KEGG) analyses, and Gene Set Enrichment Analysis (GSEA) were performed on these genes using ClusterProfiler (v3.18.1) to identify significant biological pathways or highlight biological processes with high confidence.

### Behavior tests

#### T/Y-maze test

Mice were allowed to explore all arms of the maze freely for 5 minutes. For T-maze tests, mice were placed in the central junction of a maze with arms (30 cm × 6 cm × 10 cm); for Y-maze tests, a three-arm apparatus (8 cm × 30 cm × 15 cm; 120° between arms) was used. All trials began by placing the mouse in the central starting area. Trajectories were recorded for 5 minutes using the CleverSys TopScan Lite automated video-tracking system (Clever Sys., Inc., Reston, VA, USA). An alternation was defined as consecutive entries into all three distinct arms. Alternation behavior was quantified automatically by the TopScan Lite software.

#### Elevated plus maze test

The elevated plus maze consisted of two open arms (35 cm × 5 cm) and two enclosed arms (35 cm × 5 cm) extending from a central platform (7.5 cm × 7.5 cm). Testing was conducted under quiet and dimly lit conditions. Mice were placed on the central platform of the elevated plus maze, facing an open arm. Spontaneous activity was monitored for 10 minutes, and time spent in the open arms was analyzed using CleverSys TopScan Lite (Clever Sys., Inc., Reston, VA, USA), an automated video-tracking system.

#### Open-field test

Mice were carried to the testing room at least 30 minutes prior to behavioral assessment for environmental habituation. Mice then were placed in the center of an open-field arena (50 cm × 50 cm) and allowed to explore freely for 10 minutes. Locomotor activity was recorded throughout the session, and the percentage of time spent in the central zone (24 cm × 24 cm) was quantified automatically using the CleverSys TopScan Lite video-tracking system (Clever Sys., Inc., Reston, VA, USA).

#### Novel location recognition test

The Novel Location Recognition (NLR) test assesses spatial learning, a process critically dependent on hippocampal function (19). Briefly, mice were placed in a cubic arena (45 cm × 45 cm × 45 cm) containing two identical objects and allowed to explore freely for 10 minutes (training phase). Twenty-four hours later, mice were re-exposed to two identical objects (the same objects from training), with one object relocated to a novel position equidistant from the arena walls and not in the release corner. After 10 minutes of exploration, the time spent exploring each object was recorded. A discrimination index was calculated to quantify novel location preference: (Time exploring novel location object - Time exploring familiar location object)/(Total exploration time of both objects).

#### Morris water maze test

The Morris water maze (MWM) apparatus consisted of a circular pool (110 cm diameter) maintained at 22 ± 1°C, with a hidden platform (10 cm diameter) submerged 1.0 cm below the water surface. The protocol comprised 6 consecutive days of acquisition trials followed by a probe test on day 7. During acquisition trials, mice were given 60 seconds to locate the submerged platform. Animals failing to find the platform within this period were guided to it. All mice remained on the platform for 10 seconds after each trial to reinforce spatial learning. On day 7, the platform was removed for the probe test. Time spent in the target quadrant, swimming speed and number of platform crossings were quantified using CleverSys TopScan Lite software (Clever Sys., Inc., Reston, VA, USA).

### Statistical analysis

Graphical and statistical analyses were performed in a double-blinded manner by GraphPad Prism software (GraphPad Prism software, San Diego, CA, USA, version 10.0.2). Distributed data are expressed as the mean ± SEM. The unpaired two-tailed Student’s *t*-test was used for the comparison of two groups. One-way ANOVA post-Dunnett’s multiple comparisons test was used for the comparison of more than two groups. *P* value < 0.05 was considered to be statistically significant.

## Result

### TPPU administration significantly reduces seizure severity and mitigates the associated comorbidities in a kainic acid (KA)-induced chronic epileptic mouse model

To evaluate the potential effect of TPPU on acute epileptic seizures, mice were pretreated with TPPU for 3 consecutive days, followed by intraperitoneal injection of KA at a dose of 10 mg/kg. Assessment of seizure severity using the Racine scale showed that TPPU pretreatment effectively alleviated KA-induced acute seizures (**Supplementary Figures 1A, B**). Subsequently, TPPU administration was continued for an additional 7 days. Further electrophysiological monitoring via EEG revealed that TPPU treatment significantly reduced the frequency of epileptic discharges (**Supplementary Figures 1C, D**). To evaluate the effects of TPPU on seizures and the associated comorbidities during the chronic stage, C57BL/6 mice were first pretreated with TPPU for 7 days, followed by an additional 4 weeks of TPPU treatment after KA administration (**Figure 1A**). EEG assessment showed that TPPU attenuated the frequency of epileptiform discharges, reduced spike amplitude, and improved interictal EEG parameters in KA-induced epileptic seizures (**Figures 1B, C**). The effects of TPPU on neuropsychiatric and neurobehavioral changes associated with chronic epilepsy were also evaluated in these mice. In the novel location recognition (NLR) test, TPPU significantly reversed the discrimination index deficit observed in the KA-treated group (**Figures 1D,E**). In the light/dark box test, KA-treated mice spent significantly less time in the light compartment compared with controls, whereas TPPU treatment markedly increased light-side occupancy (**Figure 1F**), suggesting an anxiolytic effect. Consistent with these findings, the elevated plus-maze test demonstrated that TPPU significantly increased both the time spent in and the number of entries into the open arms compared with KA-treated mice (**Figures 1G–I**). The Morris water maze (MWM) test was conducted to evaluate cognitive performance among the groups (**Figures 1J–M**). During the 5-day training period, KA-treated mice showed a trend toward longer escape latencies compared with both the control and TPPU-treated groups (**Figures 1J, K**). Following training, a probe test was conducted on day 6 with the platform removed. MWM analysis revealed that TPPU restored the number of target platform crossings in KA-treated mice to near-normal levels (**Figure 1L**), while swimming speeds remained comparable across groups (**Figure 1M**). These findings suggest that TPPU protects against seizures and the associated comorbidities in a chronic epilepsy mouse model.

### TPPU administration reduces neuronal apoptosis and improves synaptic function in a KA-induced chronic epileptic mouse model

Neuronal apoptosis is a well-documented pathological feature in the brains of patients with epilepsy and is closely associated with the pathogenesis of the disease. To examine whether TPPU mitigates seizure-induced neuronal apoptosis, we performed TUNEL staining combined with NeuN immunofluorescence to identify apoptotic neurons. Our results revealed a significant increase in TUNEL-positive neurons in both the cortex and hippocampal CA3 region of KA-induced chronic epileptic mice. and TPPU treatment markedly reduced the number of apoptotic neurons (**Supplementary Figures 2A–C**). Furthermore, epileptic mice exhibited downregulation of the anti-apoptotic protein Bcl-2, whereas TPPU administration led to a tendency of restore in Bcl-2 expression (**Supplementary Figures 2D, E**). These findings collectively demonstrate that TPPU exerts neuroprotective effects by ameliorating neuronal apoptosis in KA-induced chronic epilepsy.

**Figure 2:**
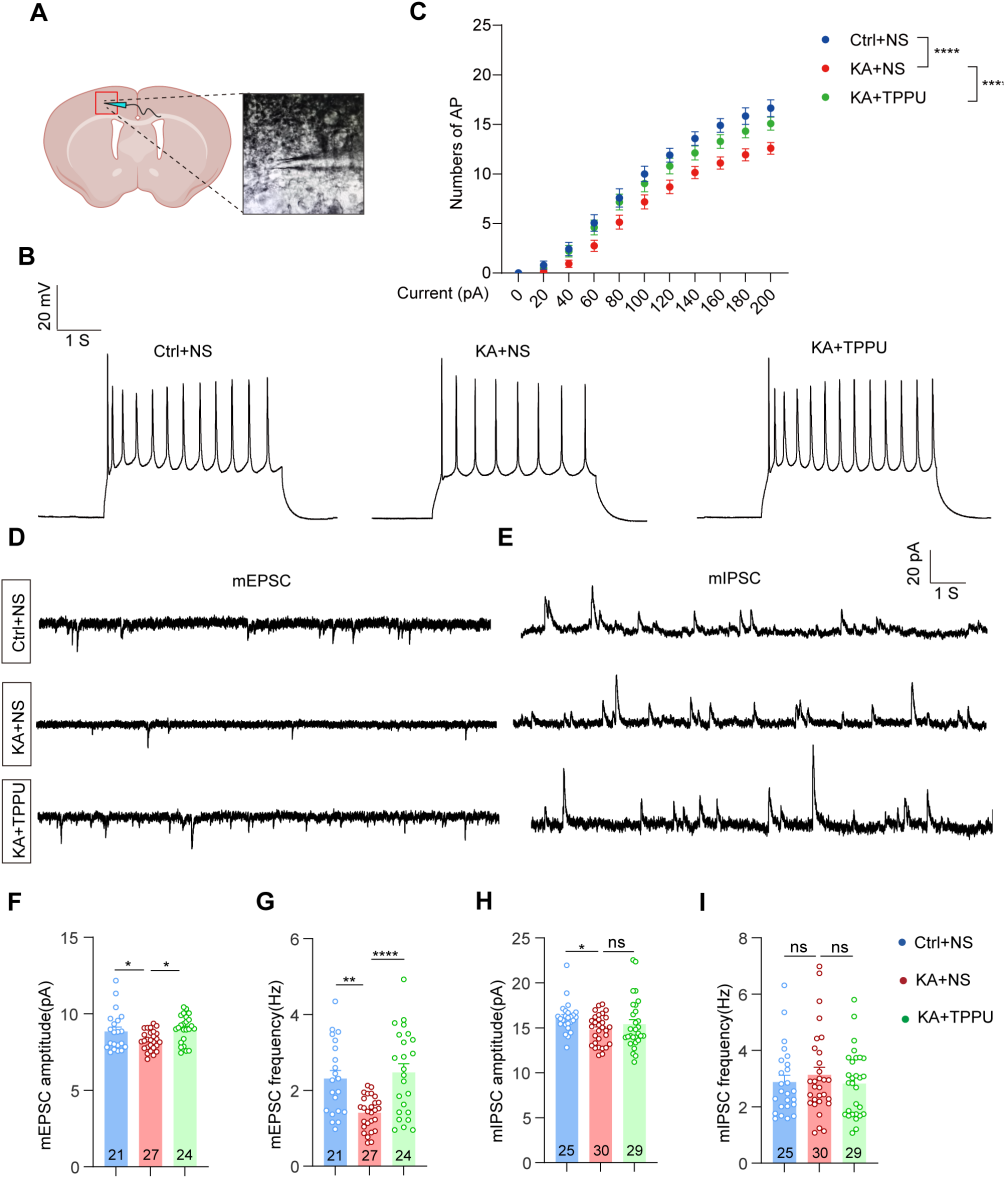
TPPU treatment enhances neuronal function and restores excitatory synaptic transmission in kainic acid-induced chronic epileptic mice. **(A)** Schematic diagram of whole-cell patch-clamp recording of frontal-temporal cortical neurons. **(B)** Representative action potential (AP) trace of whole-cell recordings under current-clamp mode from frontal-temporal cortical neurons from Ctrl+NS, KA+NS, and KA+TPPU mice. **(C)** Quantification of the number of action potentials in those mice. Ctrl+NS group: 19 neurons from n=3 mice; KA+NS group: 29 neurons from n=4 mice; KA+TPPU group: 25 neurons from n=4 mice. Data are plotted as mean ± SEM. One-way ANOVA. *p<0.05, **p<0.01, ****p<0.0001, ns: not significant. **(D)** Representative mEPSC trace from whole-cell recordings under voltage clamp mode in frontal-temporal cortical neurons of Ctrl+NS, KA+NS, and KA+TPPU mice. **(E)** Representative mIPSC trace from whole-cell recordings under voltage clamp mode in frontal-temporal cortical neurons of Ctrl+NS, KA+NS, and KA+TPPU mice. **(F, G)** The amplitude and frequency of mEPSCs in different mice were quantified; Ctrl+NS group: 21 neurons from n=3 mice; KA+NS group: 27 neurons from n=4 mice; KA+TPPU group: 24 neurons from n=4 mice. **(H, I)** Statistical chart of the amplitude and frequency of mIPSCs shown in panel B; Ctrl+NS group: n=3 mice with 25 neurons; KA+NS group: n=4 mice with 29 neurons; KA+TPPU group: n=4 mice with 28 neurons. All statistical data are expressed as mean ± standard error of the mean (SEM), and the statistical method used is One-way ANOVA. *p<0.05, **p<0.01, ****p<0.0001, ns: no statistical difference.

Given that sustained neuronal dysfunction is driven by neuronal hyperexcitability, which is essential for the generation of epileptic seizures, we next investigated how TPPU exerts its neuroprotective potential in chronic epileptic mice. Using whole-cell patch-clamp recordings from frontotemporal cortical neurons, we found that KA-induced epileptic mice exhibited significantly impaired neuronal function, as evidenced by a reduced action potential firing frequency. while TPPU treatment effectively restored action potential generation to near-normal levels (**Figures 2A–C**), demonstrating its capacity to rescue seizure-induced neuronal dysfunction.

Additionally, we assessed miniature excitatory postsynaptic currents (mEPSC) and miniature inhibitory postsynaptic currents (mIPSC) in frontotemporal cortical neurons using whole-cell patch-clamp electrophysiology. Statistical analysis of synaptic transmission revealed that neurons from chronic epileptic mice exhibited reduced mEPSC frequency and amplitude, both of which were ameliorated by TPPU treatment (**Figures 2D, F, G**). In contrast, neither the frequency nor the amplitude of mIPSC was significantly restored by TPPU treatment in these groups (**Figures 2E, H, I**). These findings suggest that TPPU enhances excitatory synaptic transmission in seizure-damaged neurons.

### TPPU treatment significantly attenuates glial activation and neuroinflammation in chronic epileptic mice

**Figure 3:**
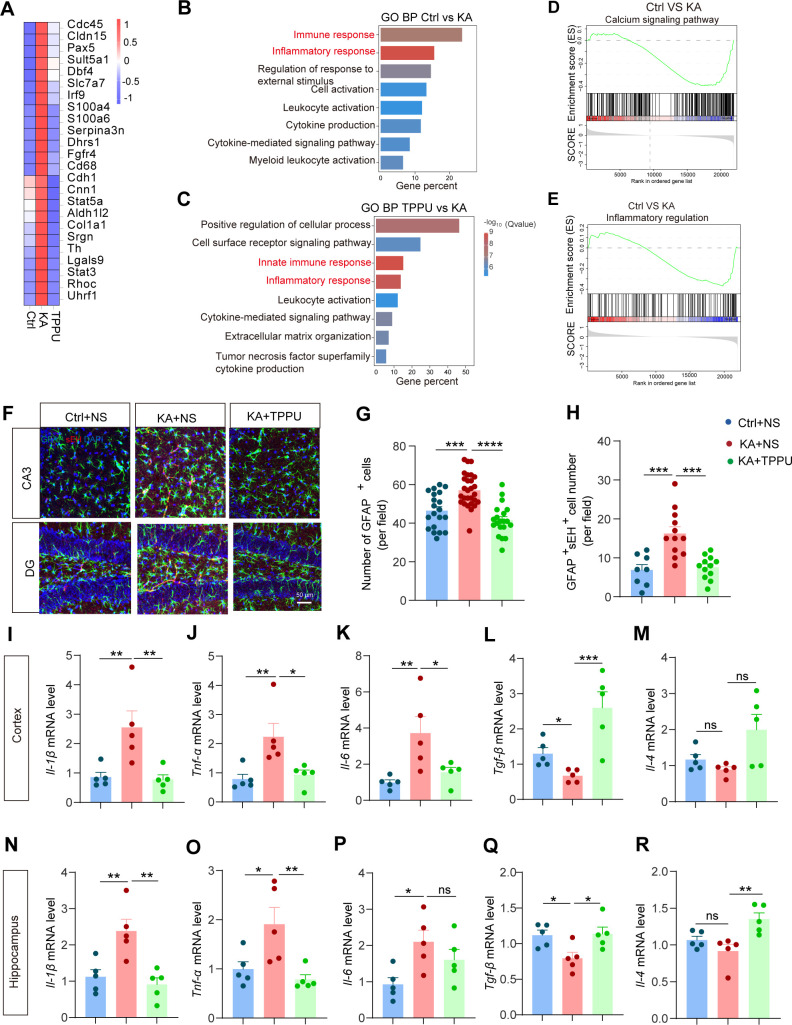
KA-induced upregulation of neuroinflammation was reversed following TPPU administration. **(A)** Heatmap analysis of differentially expressed genes in the hippocampal region of Ctrl+NS, KA+NS, and KA+TPPU mice. **(B)** GO enrichment analysis of differentially expressed genes in the Ctrl+NS vs KA+NS group. **(C)** GO enrichment analysis of differentially expressed genes in the TPPU+KA vs KA+NS group. **(D, E)** GSEA of DEGs in hippocampal samples showing enrichment with downregulated gene sets in the calcium signaling pathway in Ctrl mice versus KA treated mice **(D)** and enrichment with downregulated gene sets in the inflammatory regulation **(E)**. **(F)** Representative images of hippocampal regions from the control **(Ctrl)** or epilepsy (epilepsy) brain mice treated with or without TPPU. Slices were labeled using GFAP (green) and sEH (red) antibodies. **(G)** Statistical analysis of the number of GFAP (green) astrocytes per high-power field, n=5. **(H)** Statistical analysis of the number of GFAP (green) and sEH (red) positive (GFAP+ sEH+) astrocytes relative to GFAP+ astrocytes per high-power field. **(I–K)** Detection of the transcriptional levels of pro-inflammatory factors *IL-1β, TNF-α*, and *IL-6* in the cortex of mice from control group (Ctrl+NS), epileptic group (KA+NS), and TPPU treatment group (KA+TPPU) using RT-PCR. **(L, M)** Detection of the transcriptional levels of anti-inflammatory factors *TGF-β* and *IL-4* in the cortex of mice from control group (Ctrl+NS), epileptic group (KA+NS), and TPPU treatment group (KA+TPPU) using RT-PCR, n=5. **(N–P)** Detection of the transcriptional levels of pro-inflammatory factors *IL-1β, TNF-α*, and *IL-6* in the hippocampus of mice from control group (Ctrl+NS), epileptic group (KA+NS), and TPPU treatment group (KA+TPPU) using RT-PCR. **(Q, R)** Detection of the transcriptional levels of anti-inflammatory factors *TGF-β* and *IL-4* in the hippocampus of mice from control group (Ctrl+NS), epileptic group (KA+NS), and TPPU treatment group (KA+TPPU) using RT-PCR. Data are presented as mean ± standard error of the mean (SEM), n=5, One-way ANOVA, *p<0.05, **p<0.01, ***p<0.001, ****p<0.0001, ns, not significant.

We next performed transcriptomic profiling (RNA-Seq) to identify the molecular mechanisms by which TPPU ameliorates KA-induced epilepsy and the associated comorbidities. Heatmap visualization highlighted a marked upregulation of glial activation- related genes in KA-treated mice, which was substantially attenuated by TPPU treatment (**Figure 3A**). Gene Ontology (GO) enrichment analysis demonstrated that genes associated with the immune response and inflammatory response were significantly enriched in KA-treated epileptic mice compared with controls (**Figure 3B**), whereas TPPU treatment significantly reduced the enrichment of genes related to the innate immune response and inflammatory response (**Figure 3C**). Venn diagram shows differentially expressed genes between the three groups (**Supplementary Figure 3A**; **Supplementary Table 2**). Transcript-per-million (TPM) expression analysis corroborated these findings, showing that KA-induced overexpression of astrocyte activation markers (e.g., *Gfap*, *Cd63*, and *Serpina3n*) was significantly suppressed by TPPU. Similarly, proinflammatory genes (*Ephx4*, *Spp1*, *Vim*, and *Npy*) and nervous disease associated gene (*Rac1*, *Gria3*, *Celf2*, and *Clpp*) exhibited KA-induced upregulation that was reversed by TPPU administration (**Supplementary Figure 3B**). Gene set enrichment analysis (GSEA) revealed noteworthy enrichment in Calcium signaling pathway (**Figure 3D**) and inflammatory regulation (**Figure 3E**).

Given that TPPU is a potent sEH inhibitor that suppresses neuroinflammation, we next assessed sEH expression patterns following epilepsy induction using immunofluorescence. Compared with controls (Ctrl), sEH levels were significantly elevated in the brains of epileptic mice (KA) (**Supplementary Figures 4A–C**). Using double immunofluorescence labeling with cell-specific markers, we identified astrocytes as the primary cellular source of sEH. Notably, immunofluorescence co-localization studies revealed that sEH primarily localized to GFAP^+^ astrocytes rather than other neural cell types in the CNS after epilepsy induction (**Supplementary Figures 4A–C**), indicating enhanced astrocytic sEH expression. Furthermore, consistent with the Brain-RNAseq database (https://brainrnaseq.org/?3529285370=2053964274) (**Supplementary Figure 4D**), both quantitative real-time PCR (qRT-PCR, **Supplementary Figure 4E**) and Western blotting (**Supplementary Figures 4F, G**) analysis showed that *Ephx2* (the gene encoding sEH) expression was higher in astrocytes than in neurons and minimal expression of sEH was detected in microglia. These convergent lines of evidence - from transcriptomic databases to primary cell analyses - establish astrocytes as the predominant cellular source of sEH in the CNS. These results indicates that sEH is significantly upregulated following epilepsy induction and exhibits predominant astrocytic localization.

Building on the upregulation of sEH expression and its astrocytic localization in epileptic brains, we next investigated the effects of TPPU on astrocyte activation. Using dual immunofluorescence staining for GFAP (an astrocyte marker) and sEH, we observed that KA-induced epileptic mice exhibited significant astrogliosis compared with controls, whereas TPPU treatment reduced GFAP^+^ astrocytes to near-control levels (**Figures 3F, G**). Furthermore, analysis of hippocampal sections revealed that the sEH/GFAP colocalization in KA-treated mice was significantly reduced after TPPU treatment compared with NS (vehicle) treatment (**Figures 3F, H**). The cytochrome P450 pathway metabolizes arachidonic acid (AA) into four epoxyeicosatrienoic acid (EET) isomers (5,6-, 8,9-, 11,12-, and 14,15-EETs), which are rapidly degraded by sEH into dihydroxyeicosatrienoic acids (DHETs) (11). Notably, sEH activity is elevated in numerous neurodegenerative conditions. The inhibitory effect of TPPU on sEH was evaluated/confirmed by quantifying the ratio of 14,15-EET and 14,15-DHET in cortical and hippocampal tissues using ELISA. Notably, TPPU treatment substantially reduced 14,15-DHET levels while increased 14,15-EET levels in the cortex and hippocampus of KA-induced epileptic mice (**Supplementary Figures 5A–F**). These findings demonstrate that TPPU suppresses both epileptogenesis-associated astrocyte activation and pathological sEH expression in astrocytes.

Astrocytes and microglia are the major cell types accounting for neuroinflammation in the brain, and the activation of astrocytes typically induces microglial activation. To further validate whether TPPU affects the function and dynamics of microglia in chronic epileptic mice, we performed comprehensive immunofluorescence analyses. Quantitative Iba1^+^ staining revealed an increase in microglial density in the hippocampus and cortex of KA-induced chronic epileptic mice, and this increase was substantially reduced by TPPU treatment (**Supplementary Figures 6A, B, D**). Furthermore, by co-staining for Iba1 and CD68, a marker of phagocytic activation, we observed that the percentage of CD68-positive area was significantly higher in the epileptic group (KA+NS) than in the control group (Ctrl+NS), and this percentage was significantly reduced in the TPPU-treatment group (KA+TPPU) (**Supplementary Figures 6C, E**). Additionally, Western blot analysis of hippocampal lysates revealed that KA-induced epilepsy significantly elevated CSF1R, a protein crucial for microglial homeostasis, and this elevation was normalized by TPPU treatment (**Supplementary Figures 6F, G**). These results demonstrate that KA-induced epileptogenesis drives microglial activation, which is attenuated by TPPU treatment.

Transcriptomic profiling initially identified the suppressive effects of TPPU on astrocyte activation. To further verify whether TPPU can inhibit reactive astrocytes in an *in vitro* environment that mimics epileptic conditions we treated primary cultured astrocytes with KA, followed by treatment with TPPU for 24 h. We found that TPPU treatment significantly reduced the mRNA levels of A1 (proinflammatory) astrocyte markers *Srgn*, *Serping*, *Ugt1a1*, *Ggta1*, and *H2t23* in KA-treated astrocytes (**Supplementary Figures 7A–E**). Conversely, TPPU restored the mRNA levels of A2 (neuroprotective) astrocyte markers *Ptx3* and *Cd14* in KA-treated astrocytes, although the change in *Clcf1 was* not significant (**Supplementary Figures 7F–H**). Thus, these integrated investigations demonstrate that TPPU suppresses astrocyte activity by modulating astrocyte polarization under epileptic conditions.

Building on our transcriptomic and immunohistochemical evidence of astrocyte-mediated inflammation, we quantified cytokine expression profiles in epileptic mice in response to TPPU treatment using RT-PCR. Compared with controls, TPPU treatment significantly attenuated the KA-induced elevations of the proinflammatory cytokines *IL-1β, TNF-α*, and *IL-6* in both cortical and hippocampal tissues (**Figures 3I–K, N–P**). Furthermore, the anti-inflammatory cytokines were also evaluated in these mice. TPPU restored *TGF-β* mRNA levels (**Figures 3L, Q**), while *IL-4* levels remained significantly elevated or showed a trend toward further increase following TPPU treatment (**Figures 3M, R**). We also examined the expression of additional cytokines, including *Ccl2*, *Ccl4*, *Ccl5*, *Ccl8*, and *Ccl11*. In the hippocampus, KA treatment significantly upregulated *Ccl2* and *Ccl5*, while TPPU downregulated their expression. Although no significant differences were observed for the other cytokines, they all exhibited a trend toward upregulation following KA treatment, which was attenuated by TPPU (**Supplementary Figure 8**).

Collectively, these results implicate that TPPU exerts neuroprotective effects, at least in part, through the modulation of astrocyte reactivity and inflammatory signaling pathways.

### TPPU protects against neuroinflammation by inhibiting the Akt/mTOR signaling pathway in astrocytes

**Figure 4:**
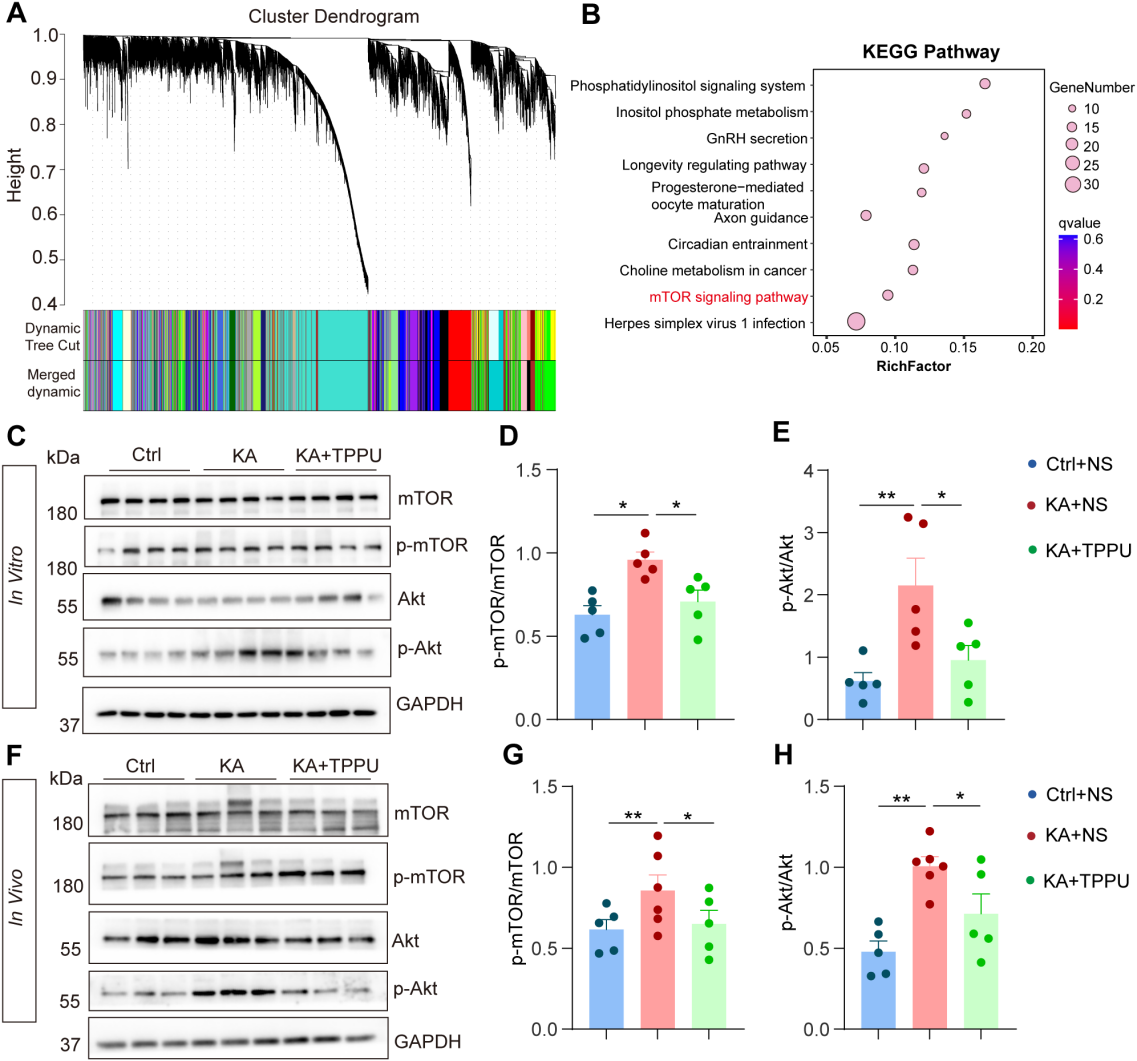
TPPU inhibits the Akt/mTOR signaling pathway in astrocytes and chronic epileptic mice. **(A)** WGCNA dendrogram of all differentially expressed genes (DEGs) from mouse hippocampus treated with or without KA and/TPPU. **(B)** KEGG pathway enrichment analysis on WGCNA black module, mTOR signaling pathway was significantly enriched, red highlighted. **(C)** Astrocytes were treated with KA (100 μM) and TPPU (100 μM) for 24 hours. Representative images of Western blot for p-Akt/Akt and p-mTOR/mTOR in astrocytes are shown. **(D)** Protein levels of p-mTOR/mTOR were quantified by densitometry and are presented as ratios to GAPDH. **(E)** Protein levels of p-Akt/Akt were quantified by densitometry and are presented as ratios to GAPDH. **(F)** Representative images of Western blot for p-Akt/Akt, and p-mTOR/mTOR in chronic epileptic mouse brains treated with or without TPPU are shown. **(G)** Protein levels of p-mTOR/mTOR in those mouse brains were quantified by densitometry and are presented as ratios to GAPDH. **(H)** Protein levels of p-Akt/Akt in those mouse brains were quantified by densitometry and are presented as ratios to GAPDH. Data are presented as mean ± standard error of the mean (SEM), n=5, One-way ANOVA, *p<0.05, **p<0.01.

Hyperactivation of the Akt/mTOR signaling cascade is consistently observed across multiple epileptic models (e.g., kainate, pilocarpine), suggesting a central mechanistic role in ictogenesis (20–22). To further investigate the molecular mechanisms underlying the anti-epileptic effects of TPPU, we performed weighted gene co-expression network analysis (WGCNA) on RNA-seq data from mice treated with or without KA and/or TPPU. WGCNA identified a set of hierarchical clusters (**Figure 4A**), We performed KEGG pathway enrichment analysis on each WGCNA module, for the turquoise module, the most significantly enriched pathway was the PI3K-Akt signaling pathway (**Supplementary Figure 9**), in addition, the black module showed specific enrichment of the mTOR signaling pathway (**Figure 4B**), these results indicate that TPPU may regulate the two interconnected signaling cascades in a module-specific manner. Therefore, we next examined the phosphorylation levels of Akt, and mTOR in primary astrocytes treated with KA and/or TPPU *in vitro* by Western blotting. TPPU treatment markedly reduced the KA-induced phosphorylation of Akt/mTOR (**Figures 4C–E**). Activation of the Akt/mTOR pathway was further confirmed in the hippocampal tissue of KA-induced chronic epileptic mice, where TPPU downregulated the levels of p-Akt, and p-mTOR (**Figures 4F–H**). These results indicate that TPPU protects against neuroinflammation by inhibiting the Akt/mTOR signaling pathway in astrocytes of chronic epileptic mice.

## Discussion

The spectrum of comorbidities in epilepsy encompasses a diverse range of psychiatric, cognitive, and somatic disorders that collectively affect approximately 60% of patients with epilepsy (23). These comorbid conditions significantly impair quality of life and may exacerbate seizure frequency through several shared pathophysiological mechanisms. Current evidence implicates neuroinflammation, hypothalamic-pituitary-adrenal (HPA) axis dysregulation, neuronal network hyperexcitability, and progressive neuronal damage as key interrelated processes underlying the complex interplay between epilepsy and its comorbidities (5, 24, 25). The present findings establish TPPU as a multifunctional therapeutic candidate capable of concurrently targeting both seizures and the associated comorbidities. This study not only highlights the therapeutic potential of TPPU but also provides a mechanistic foundation for its dual efficacy in seizure control and comorbidity alleviation.

To investigate the potential mechanisms underlying the therapeutic effects of TPPU in epilepsy, we performed whole-transcriptome RNA sequencing (RNA-Seq) on mouse hippocampal tissues. Our results suggest that astrocytes and inflammatory responses play a key role in the development of epilepsy, and that TPPU may exert neuroprotective effects by modulating astrocytes. Emerging evidence underscores the critical role of inflammatory mechanisms in regulating neuronal function, with dysregulated neuroinflammation contributing significantly to both the pathogenesis of epilepsy and its associated comorbidities (26). Abnormal inflammatory signaling can cause synaptic dysfunction and contributes to the development of related neurological disorders (27). Although neurons are the principal executors of seizure activity, glial cells—particularly astrocytes and microglia—play a prominent role in mediating the neuroinflammation that regulates or triggers seizures. These glial cells can sense aberrant neuronal activity and respond to pathological changes by becoming activated and releasing various pro-inflammatory factors, which in turn exacerbate abnormal neuronal discharges and promote the progression of epilepsy (28, 29). Seizures and neuroinflammation mutually reinforce each other, forming a vicious cycle that facilitates the development of refractory epilepsy. Studies have demonstrated that pro-inflammatory factors such as TNF-α, IL-1β, and IL-6 are significantly elevated in the serum of patients with various epileptic syndromes following seizures, and the levels of the inflammatory cytokines IL-1β, IL-6, IL-10, and TNF-α are increased in the brain tissue of rats after status epilepticus (30–32).

sEH is a key enzyme that regulates inflammatory responses in the brain. In the central nervous system, it hydrolyzes EETs into pro-inflammatory metabolites, such as 14,15-DHET. As a potent sEH inhibitor, TPPU reduces the generation of 14,15-DHET by maintaining higher levels of EETs, thereby exerting anti-inflammatory and neuroprotective effects (33). Our findings identify astrocytic sEH as a central regulator bridging epoxyeicosatrienoic acid (EET) metabolic dysfunction with neuroinflammatory cascades in epilepsy. The increased expression of sEH under pathological conditions in epilepsy may lead to decreased EET levels and elevated 14,15-DHET levels, thereby exacerbating neuroinflammation and neuronal hyperexcitability. Therefore, the upregulation of sEH in epilepsy may not only aggravate the inflammatory response but also promote the progression of epilepsy through neuronal damage. Consistent with previous studies reporting that sEH is primarily expressed in astrocytes in the CNS (34), this study also found that astrocytes, but not neurons or microglia, are the predominant cellular source of sEH in the CNS. This selective expression pattern suggests that astrocytic sEH may play a privileged role in modulating EET-mediated neuroprotection. We found that the increased localization of sEH in astrocytes was significantly reversed by TPPU treatment, indicating that TPPU effectively inhibits sEH activity and thereby alleviate glial cell-mediated neuroinflammatory responses.

During the pathological process of epilepsy, the activation of astrocytes and microglia is a major driver of neuroinflammation and neuronal damage (35). We found that TPPU administration reduces neuronal apoptosis and improves synaptic function in a KA-induced chronic epileptic model, which may be explained by the ability of astrocytes to maintain normal neuronal excitability through the regulation of glutamate concentrations in the synaptic cleft. In the CNS, astrocytes are the most abundant glial cell type. They perform diverse functions, including providing growth factors, regulating the balance of ions and neurotransmitters, and maintaining brain homeostasis (36). Abnormally activated astrocytes may lead to reduced glutamate uptake, accumulation of glutamate in the synaptic cleft, and neuronal hyperexcitability, which can trigger seizures (37). In the CNS, astrocytes and microglia closely interact to regulate inflammatory responses. Microglia—the CNS’s specialized innate immune cells—serve as critical mediators of neuroinflammatory priming and epileptogenesis through their dynamic surveillance and immune regulatory functions (38, 39). Our findings also demonstrate that the ability of TPPU to restore microglial homeostasis represents a novel strategy for multi-mechanistic epilepsy therapy, simultaneously targeting both inflammatory drivers and neuronal network destabilization. This microglial stabilization complements the established effects of TPPU on astrocytic sEH, creating a comprehensive glial-targeted therapeutic approach.

Repeated seizures in epilepsy are often accompanied by a chronic inflammatory state, and the sustained elevation of pro-inflammatory factors can worsen the abnormal excitability of neural networks. Therefore, modulating inflammatory factors helps restore homeostasis in the microenvironment surrounding the epileptic focus, reducing neuronal damage and abnormal excitability (25). The observed cytokine modulation reveals the capacity of TPPU to reprogram the immune microenvironment of the epileptic brain—simultaneously quenching detrimental neuroinflammation (via IL-1β/TNF-α/IL-6 suppression) and amplifying endogenous protective mechanisms (through TGF-β upregulation). This dual action may underlie its superior neuroprotection compared with conventional anti-inflammatory approaches. Based on our findings, TPPU demonstrates significant anti-inflammatory and neuroprotective effects in KA-induced epileptic mice, with astrocytes serving as the primary cellular mediators. To further elucidate the underlying molecular mechanisms, we investigated how TPPU modulates astrocyte function. Our findings reveal TPPU as a master regulator of astrocyte polarization, acting by simultaneously: (1) suppressing neurotoxic A1 transformation through complement pathway inhibition, and (2) enhancing neuroprotective A2 functions via glutamate transporter stabilization. This dual action breaks the vicious cycle of neuroinflammation and hyperexcitability in epilepsy.

Astrocytes undergo functional polarization into two distinct states: the neurotoxic A1 phenotype and the neuroprotective A2 phenotype. Studies have shown that A1-type astrocytes are increased in various mouse models of epilepsy and can exacerbate neuronal damage after seizures, and that inhibiting the transformation of astrocytes into the A1 phenotype can mitigate the severity of epilepsy and neuronal damage (40–43). Conversely, A2-type astrocytes play a relatively beneficial role in epilepsy, and promoting the activity of A2-type astrocytes can enhance glutamate uptake and reduce extracellular glutamate levels, potentially protecting neurons from excitotoxic effects (44, 45). In this study, our transcriptome data also showed that TPPU inhibited the mRNA expression of A1-type astrocyte markers *Srgn, Serping, Ugt1a1, Ggta1*, and *H2t23*, while promoting the mRNA expression of A2-type astrocyte markers *Ptx3* and *Cd14*.

Hyperactivation of the Akt/mTOR signaling cascade is consistently observed across multiple epileptic models (e.g., kainate, pilocarpine), suggesting a central mechanistic role in ictogenesis. Activation of the Akt/mTOR pathway increases neuronal excitability and synaptic remodeling, which heightens susceptibility to seizures and is associated with structural abnormalities in the brain (20). Studies have shown that animal models with deletion of mTOR negative regulators exhibit characteristic epileptic phenotypes, with neurons displaying abnormal dendritic structures and aberrant synaptic growth (21). Overactivation of mTOR is also closely associated with neuroinflammation, characterized by elevated levels of pro-inflammatory factors, including IL-1β and TNF-α, which can exacerbate neuronal damage via the Akt/mTOR pathway (22). It has been demonstrated that inhibiting mTOR activity in glial cells reduces the frequency of seizures, indicating that this pathway plays a significant role in the glial dysfunction underlying epilepsy (46). Rapamycin and other mTOR inhibitors have been shown to alleviate the frequency and severity of seizures by inhibiting the mTOR signaling pathway, supporting the regulation of the Akt/mTOR pathway as an important therapeutic target for epilepsy (47).

We found that TPPU significantly inhibited the astrocytic Akt/mTOR pathway in chronic epilepsy. Consistent with our findings, the sEH/COX-2 dual inhibitor PTUPB has been shown to enhance hepatocyte autophagy and delay hepatocyte senescence by inhibiting the PI3K/AKT/mTOR pathway through Sirt1, thereby ameliorating non-alcoholic fatty liver disease in mice (48). TPPU restores impaired autophagic flux in cardiac fibroblasts by inhibiting mTOR activity, thereby attenuating chronic ethanol-induced cardiac fibrosis in mice (49). The sEH inhibitor AUDA regulates cell death-related signaling pathways by inhibiting TNF-α-induced mTOR phosphorylation, thereby suppressing the proliferation and migration of human aortic smooth muscle cells (50). Given the complexity of the Akt/mTOR signaling pathway and its role as a “hub” in regulating epileptogenesis, we propose that TPPU exert its anticonvulsant and comorbidity-alleviating effects by inhibiting the activation of this pathway.

Our work in the KA model complements existing literature and strengthens the evidence for the broad applicability of sEH inhibition across different epileptogenic etiologies. Although our preclinical data establish the multi-mechanistic benefits of TPPU, clinical translation requires (1) target engagement validation in higher species, (2) comprehensive safety profiling, and (3) demonstration of synergistic effects with existing therapies. This study indicates that TPPU administered in the early stages of epileptogenesis has the potential to reduce seizure severity while simultaneously preventing the development of neuropsychiatric comorbidities. This study provides a rationale for TPPU as a preventive strategy for patients at high risk of developing epilepsy and its associated comorbidities.

### Limitations of the study

We recognize that this study has several limitations. First, although preliminary assessments revealed no overt behavioral or physiological changes in naive mice treated with TPPU at the dose used, a Ctrl+TPPU group (no KA) was not included in the main experimental cohorts. Second, the TPPU pretreatment paradigm does not address its efficacy as a treatment for comorbidities in chronic, established epilepsy. Future studies employing a therapeutic design, in which TPPU administration is initiated after epilepsy is fully established, are warranted to directly evaluate its potential to prevent comorbidities in chronic epilepsy. Third, we did not employ specific interventions (such as Akt/mTOR pathway inhibitors, knockout, or mutation of Akt/mTOR pathway-related molecules) for functional validation, which should be fully addressed in future studies. Fourth, the control group (Ctrl+NS and KA+NS) in the current study received normal saline rather than the vehicle solution (10% DMSO, 40% PEG400, and 50% normal saline) used to dissolve TPPU. We acknowledge that this represents a limitation in experimental design, as the inclusion of identical vehicle-treated control groups would have more rigorously excluded any potential vehicle-related effects. Although previous studies (16, 17, 51) from our group have routinely used PEG400 in saline as the vehicle control for TPPU and have consistently observed no significant effects of the vehicle on behavioral performance, seizure susceptibility, or molecular endpoints, future studies will incorporate complete vehicle control groups as recommended to further strengthen the experimental rigor.

## Data Availability

The datasets presented in this study can be found in online repositories. The names of the repository/repositories and accession number(s) can be found in the article/**Supplementary Material**.

## Supplementary material

Supplementary Table 1 Information of primer sequences for qRT-PCR experiments.

Supplementary Table 2 Differentially expressed genes between the comparisons: KA+NS vs. Ctrl+NS, KA+TPPU vs. KA+NS, and KA+TPPU vs. Ctrl+NS.

Supplementary Figure 1 TPPU treatment significantly ameliorates seizure severity in acute epileptic seizure. (A) Schematic time line of TPPU administration in KA-induced acute epileptic mouse model. (B) Racine score assessment from different groups. (C) Representative EEG recordings from different groups. (D) Statistical analysis of epileptiform discharge frequency (spike number) in different groups during 1 hr recording.

Supplementary Figure 2 TPPU improves neuronal apoptosis in the cortex and hippocampus of chronic epileptic mice. (A, B) Representative images of apoptotic neurons in mouse brain labelled by TUNEL and NeuN immunofluorescent staining. (C) Quantification of TUNEL-positive and NeuN-positive (Tunel+NeuN+) neurons per high-power field from those brain slices. (D) Representative Western blot images of Bcl-2 expression in the hippocampus of Ctrl+NS, KA+NS, and KA+TPPU mice. (E) Bcl-2 levels were quantified by densitometry and are presented as ratios to GAPDH. Data are plotted as mean ± SEM. n =5, One-way ANOVA. *p<0.05, **p<0.01, ***p<0.001, ****p<0.0001; ns, not significant.

Supplementary Figure 3 RNA-Sequencing shows the differentially expressed genes between three groups. (A) Venn diagram shows differentially expressed genes between the comparisons: KA+NS vs. Ctrl+NS, KA+TPPU vs. KA+NS, and KA+TPPU vs. Ctrl+NS. (B) Transcript-per-million (TPM) expression analysis of astrocyte activation markers (e.g., Gfap, Cd63, and Serpina3n), proinflammatory genes (Ephx4, Spp1, Vim, and Npy) and nervous disease associated gene (Rac1, Gria3, Celf2, and Clpp).

Supplementary Figure 4 Soluble epoxide hydrolase (sEH) is predominantly expressed in astrocytes in the CNS and is increased following epilepsy induction. (A) Representative immunofluorescent images of sEH localization in the control (Ctrl) or epilepsy (KA) brain slices labelled using GFAP (green) and sEH (red) antibodies. (B) Representative immunofluorescent images of sEH localization in the control (Ctrl) or epilepsy (KA) brain slices labelled using Iba1 (green) and sEH (red) antibodies. (C) Representative immunofluorescent images of sEH localization in the control (Ctrl) or epilepsy (KA) brain slices labelled using NeuN (green) and sEH (red) antibodies. (D) The transcriptional levels of the sEH coding gene mus-Ephx2 in different cell types in the brain from the brain-seq database. (E) Detection of the transcriptional levels of Ephx2 in primary cultured neurons, astrocytes, and microglia using quantitative real-time PCR. (F) Representative images of Western blotting for sEH in different cell types are shown. (G) Protein levels of sEH were quantified by densitometry and are presented as ratios to β-actin. Data are presented as mean ± standard error of the mean (SEM), n=3, One-way ANOVA, **p<0.01, ***p<0.001.

Supplementary Figure 5 TPPU inhibits the activity of sEH in chronic epileptic mice. (A) The levels of 14,15-DHET, a sEH catalytic product, in the cortex of mice from the control group (Ctrl+NS), epileptic group (KA+NS), and TPPU treatment group (KA+TPPU) were detected by ELISA. (B) The levels of 14,15-EET in the cortex of mice from the control group (Ctrl+NS), epileptic group (KA+NS), and TPPU treatment group (KA+TPPU) were detected by ELISA. (C) The ratio of 14,15-EET/14,15 DHET in the cortex of mice from the control group (Ctrl+NS), epileptic group (KA+NS), and TPPU treatment group (KA+TPPU). (D) The levels of 14,15-DHET, a sEH catalytic product, in the hippocampus of mice from the control group (Ctrl+NS), epileptic group (KA+NS), and TPPU treatment group (KA+TPPU) were detected by ELISA. (E) The levels of 14,15-EETin the hippocampus of mice from the control group (Ctrl+NS), epileptic group (KA+NS), and TPPU treatment group (KA+TPPU) were detected by ELISA. (F) The ratio of 14,15-EET/14,15 DHET in the hippocampus of mice from the control group (Ctrl+NS), epileptic group (KA+NS), and TPPU treatment group (KA+TPPU). Data are presented as mean ± standard error of the mean (SEM), n=5, One-way ANOVA, *p<0.05, **p<0.01.

Supplementary Figure 6 TPPU inhibits microglial activation in chronic epileptic mice. (A) Representative images of hippocampal regions from the control (Ctrl) or epilepsy (epilepsy) brain mice treated with or without TPPU. Slices were labelled using Iba1 (green) and CD68 (red) antibodies. (B) The number of Iba1 (green) microglia per high-power field in the hippocampus of those mice. (C) The percentage of Iba1 (green) and CD68 (red) positive (GFAP+ sEH+) microglia relative to Iba1+ microglia per high-power field in the hippocampus of those mice. (D) The number of Iba1 (green) microglia per high-power field in the cortex of those mice. (E) The percentage of Iba1 (green) and CD68 (red) positive (GFAP+ sEH+) microglia relative to Iba1+ microglia per high-power field in the cortex of those mice. (F) Representative images of Western blot for CSF1R in the hippocampal tissue of Ctrl+NS, KA+NS, and KA+TPPU mice. (G) Protein levels of CSF1R were quantified by densitometry and are presented as ratios to GAPDH. Data are presented as mean ± standard error of the mean (SEM), n=4-6, One-way ANOVA, *p<0.05, **p<0.01, ***p<0.001, ****p<0.0001.

Supplementary Figure 7 TPPU inhibits the activation of reactive astrocytes. (A–E) Primary astrocytes were treated with KA (100 μM) and TPPU (100 μM) for 24 hours, and the transcriptional expression levels of A1 reactive astrocyte-related markers Srgn, Serping, Ugt1a1, Ggta1, and H2t23 were assessed using quantitative real-time PCR. (F–H) The transcriptional expression levels of A2 reactive astrocyte-related markers Ptx3, Cd14, and Clcf1 were measured by quantitative real-time PCR. Data are presented as mean ± standard error of the mean (SEM), n=3, One-way ANOVA, *p<0.05, **p<0.01, ***p<0.001, ****p<0.0001, ns: no statistical difference.

Supplementary Figure 8 TPPU inhibits neuroinflammation in chronic epileptic mice. (A–E) Detection of the transcriptional levels of cytokine Ccl2, Ccl4, Ccl5, Ccl8 and Ccl11 in the cortex of mice from control group (Ctrl+NS), epileptic group (KA+NS), and TPPU treatment group (KA+TPPU) using RT-PCR. (F–J) Detection of the transcriptional levels of cytokine Ccl2, Ccl4, Ccl5, Ccl8 and Ccl11 in the hippocampus of mice from control group (Ctrl+NS), epileptic group (KA+NS), and TPPU treatment group (KA+TPPU) using RT-PCR. N=5. One-way ANOVA, *p<0.05, **p<0.01, ns, not significant.

Supplementary Figure 9 KEGG pathway enrichment analysis on WGCNA turquoise module. PI3K-Akt signaling pathway was significantly enriched, red box highlighted.
