# Supplementary material for: TPPU protects against seizures and seizure-associated comorbidities by inhibiting the Akt/mTOR signaling pathway in KA-induced convulsant mice
Source: Front Immunol. 2026 Jun 8;17:1850303. doi: 10.3389/fimmu.2026.1850303 (PMC13283869; doi:10.3389/fimmu.2026.1850303)
Supplement: Supplementary Table 1 — Information of primer sequences for qRT-PCR experiments. [file Table1.docx]

**Supplementary Table 1.Information of primer sequences for qRT-PCR experiments.**

|  | GenBank | Forward primer (5’−3’) | Reversed primer (5’−3’) |
| --- | --- | --- | --- |
| *Actb* | NM_11461 | TCTTGGGTATGGAATCCTGTGGCA | TCTTGGGTATGGAATCCTGTGGCA |
| *mIl-1β* | NM_16176 | GCCACCTTTTGACAGTGATGAG | ATGTGCTGCTGCGAGATTTG |
| *mTnf-α* | NM_21926 | GTCTACTGAACTTCGGGGTGAT | CTGAGTGTGAGGGTCTGGGC |
| *mIl-6* | NM_16193 | CTCATTCTGCTCTGGAGCCC | CAACTGGATGGAAGTCTCTTGC |
| *mTgf-β* | NM_21812 | CCACAAACAGTGGCGGC | AAACACTGTAATGCCTTCGCC |
| *mIl-4* | NM_16189 | GGTCTCAACCCCCAGCTAGT | GCCGATGATCTCTCTCAAGTGAT |
| *mSrgn* | NM_19073 | CTCGCCTTCGTCCTGGTTT | CCTCGATGCAGTTCGCAAAAA |
| *mSerping1* | NM_12258 | TAGAGCCTTCTCAGATCCCGA | ACTCGTTGGCTACTTTACCCA |
| *mGgta1* | NM_14594 | GGTGGTTCCCAAGCTGGTTTA | CGGGCGGTTCTTTGGATTGA |
| *mUgt1a1* | NM_394436 | GCTTCTTCCGTACCTTCTGTTG | GCTGCTGAATAACTCCAAGCAT |
| *mH2-t23* | NM_15040 | ACAGTCCCGACCCAGAGTAG | CCACGTAGCCGACAATGATGA |
| *mPtx3* | NM_19288 | CCTGCGATCCTGCTTTGTG | GGTGGGATGAAGTCCATTGTC |
| *mCd14* | NM_12475 | CTCTGTCCTTAAAGCGGCTTAC | GTTGCGGAGGTTCAAGATGTT |
| *mClcf1* | NM_ 56708 | GACTCGTGGGGGATGTTAGC | CTAAGCTGCGGAGTTGATGCT |
| *mCcl2* | NM_011333 | AACTGCATCTGCCCTAAGGT | AGGCATCACAGTCCGAGTCA |
| *mCcl4* | NM_013652 | GAAACAGCAGGAAGTGGGAG | CATGAAGCTCTGCGTGTCT |
| *mCcl5* | NM_013653 | TGCTGCTTTGCCTACCTCTC | TTGGCACACACTTGGCGGTT |
| *mCcl8* | NM_021443 | AGGGATTGAGAGGACGCTAG | GGTGACTGGAGCCTTATCTG |
| *mCcl11* | NM_011329 | AGCTAGTCGGGAGAGCCTAC | AAGGAAGTGACCGTGAGCAG |
