# Supplementary figures and images for: TPPU protects against seizures and seizure-associated comorbidities by inhibiting the Akt/mTOR signaling pathway in KA-induced convulsant mice
Source: Front Immunol. 2026 Jun 8;17:1850303. doi: 10.3389/fimmu.2026.1850303 (PMC13283869; doi:10.3389/fimmu.2026.1850303)

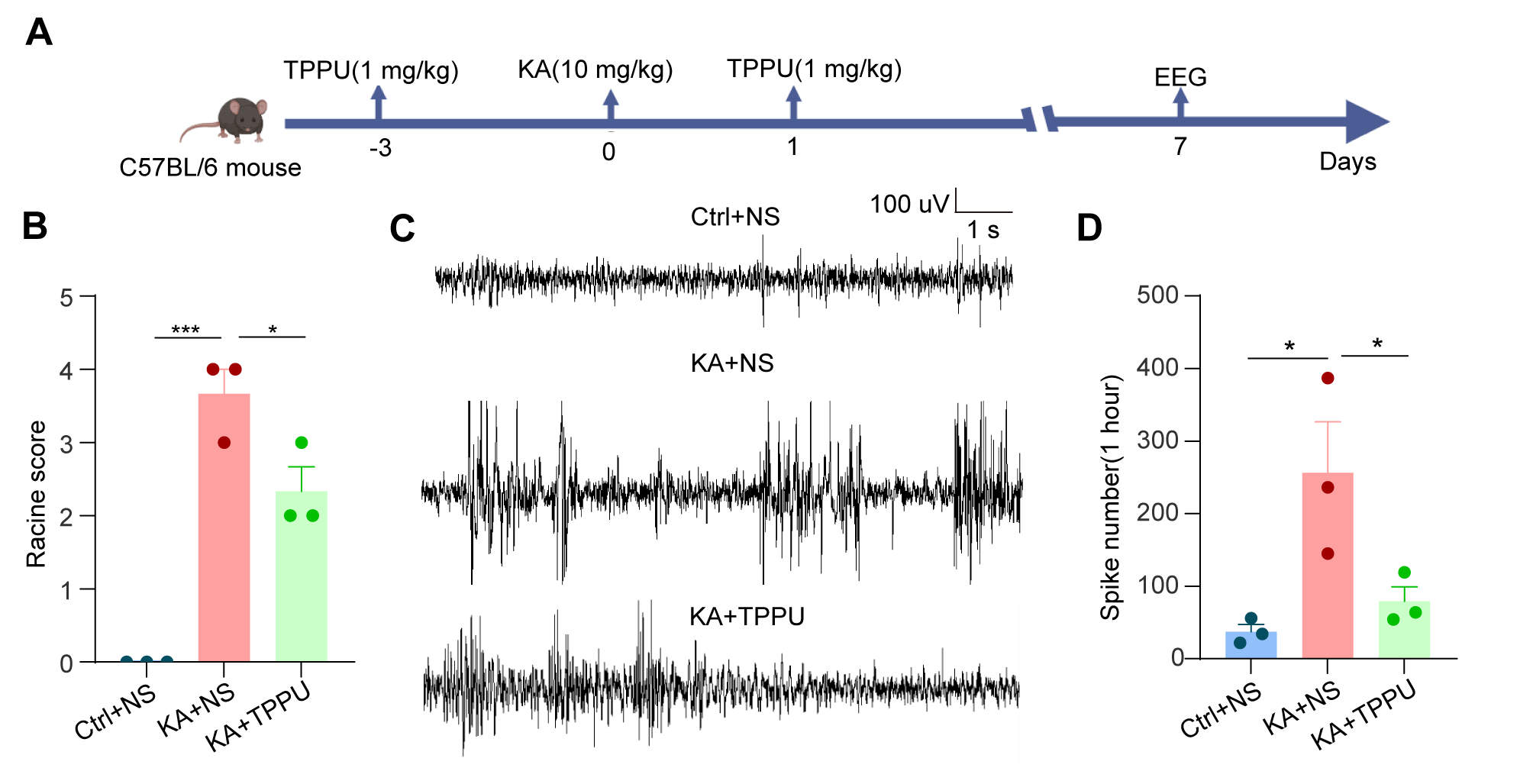

Supplement: Supplementary Figure 1 — TPPU treatment significantly ameliorates seizure severity in acute epileptic seizure. (A) Schematic time line of TPPU administration in KA-induced acute epileptic mouse model. (B) Racine score assessment from different groups. (C) Representative EEG recordings from different groups. (D) Statistical analysis of epileptiform discharge frequency (spike number) in different groups during 1 hr recording. [file Image1.tif]

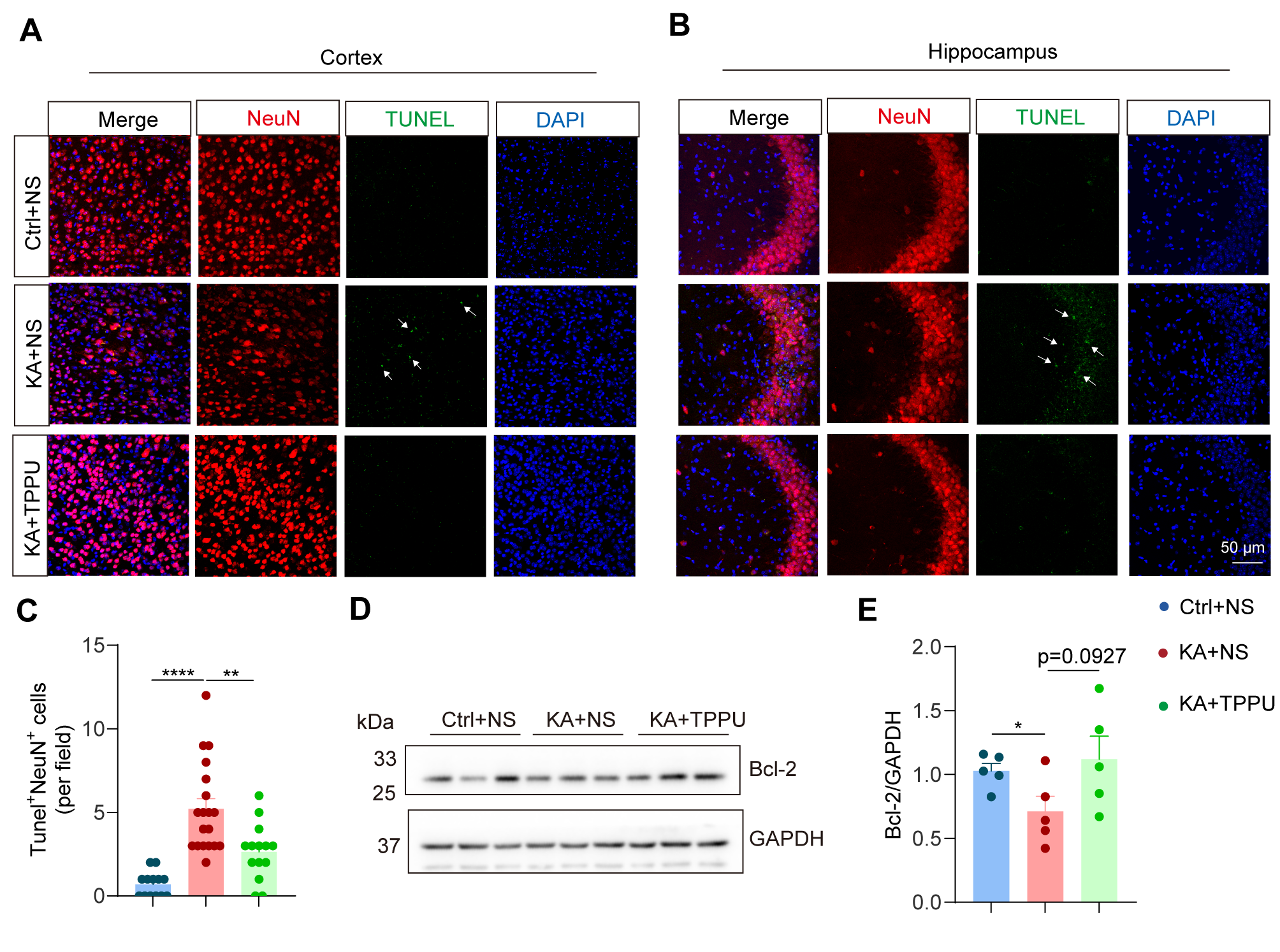

Supplement: Supplementary Figure 2 — TPPU improves neuronal apoptosis in the cortex and hippocampus of chronic epileptic mice. (A, B) Representative images of apoptotic neurons in mouse brain labelled by TUNEL and NeuN immunofluorescent staining. (C) Quantification of TUNEL-positive and NeuN-positive (Tunel+NeuN+) neurons per high-power field from those brain slices. (D) Representative Western blot images of Bcl-2 expression in the hippocampus of Ctrl+NS, KA+NS, and KA+TPPU mice. (E) Bcl-2 levels were quantified by densitometry and are presented as ratios to GAPDH. Data are plotted as mean ± SEM. n =5, One-way ANOVA. *p<0.05, **p<0.01, ***p<0.001, ****p<0.0001; ns, not significant. [file Image2.tif]

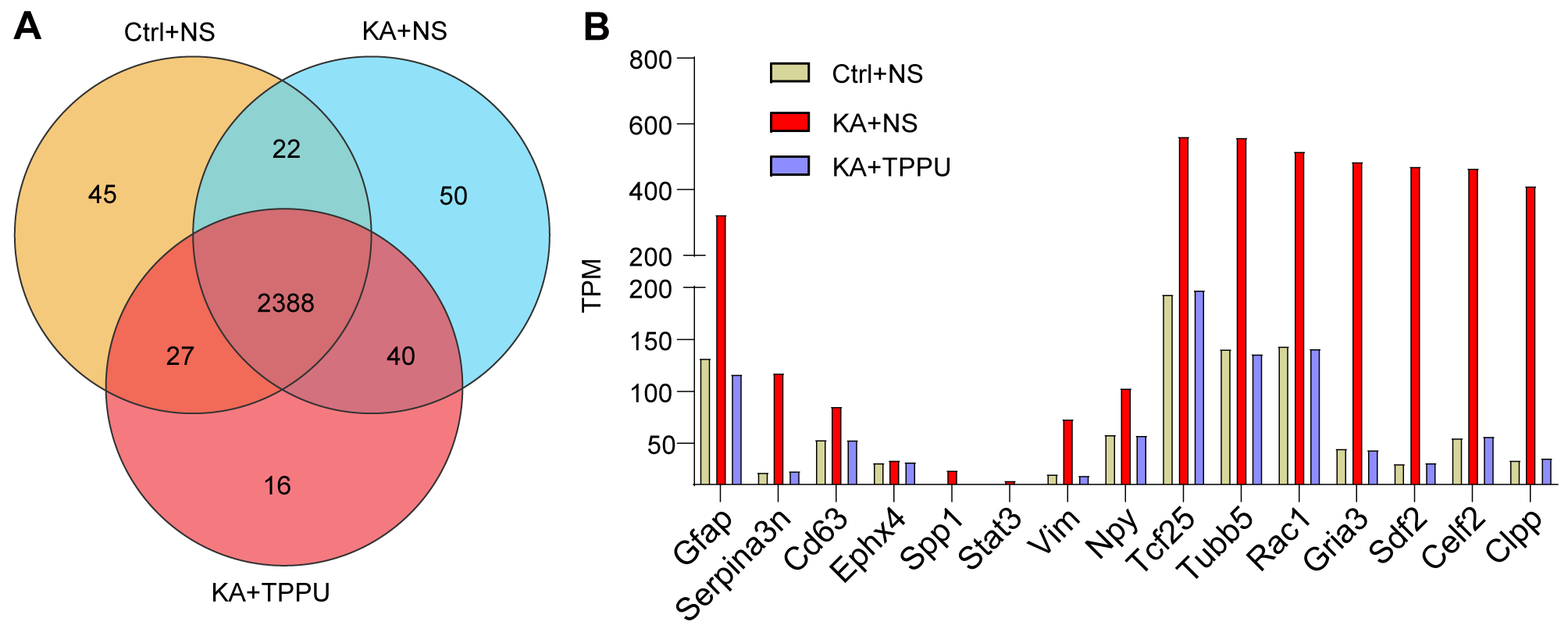

Supplement: Supplementary Figure 3 — RNA-Sequencing shows the differentially expressed genes between three groups. (A) Venn diagram shows differentially expressed genes between the comparisons: KA+NS vs. Ctrl+NS, KA+TPPU vs. KA+NS, and KA+TPPU vs. Ctrl+NS. (B) Transcript-per-million (TPM) expression analysis of astrocyte activation markers (e.g., Gfap, Cd63, and Serpina3n), proinflammatory genes (Ephx4, Spp1, Vim, and Npy) and nervous disease associated gene (Rac1, Gria3, Celf2, and Clpp). [file Image3.tif]

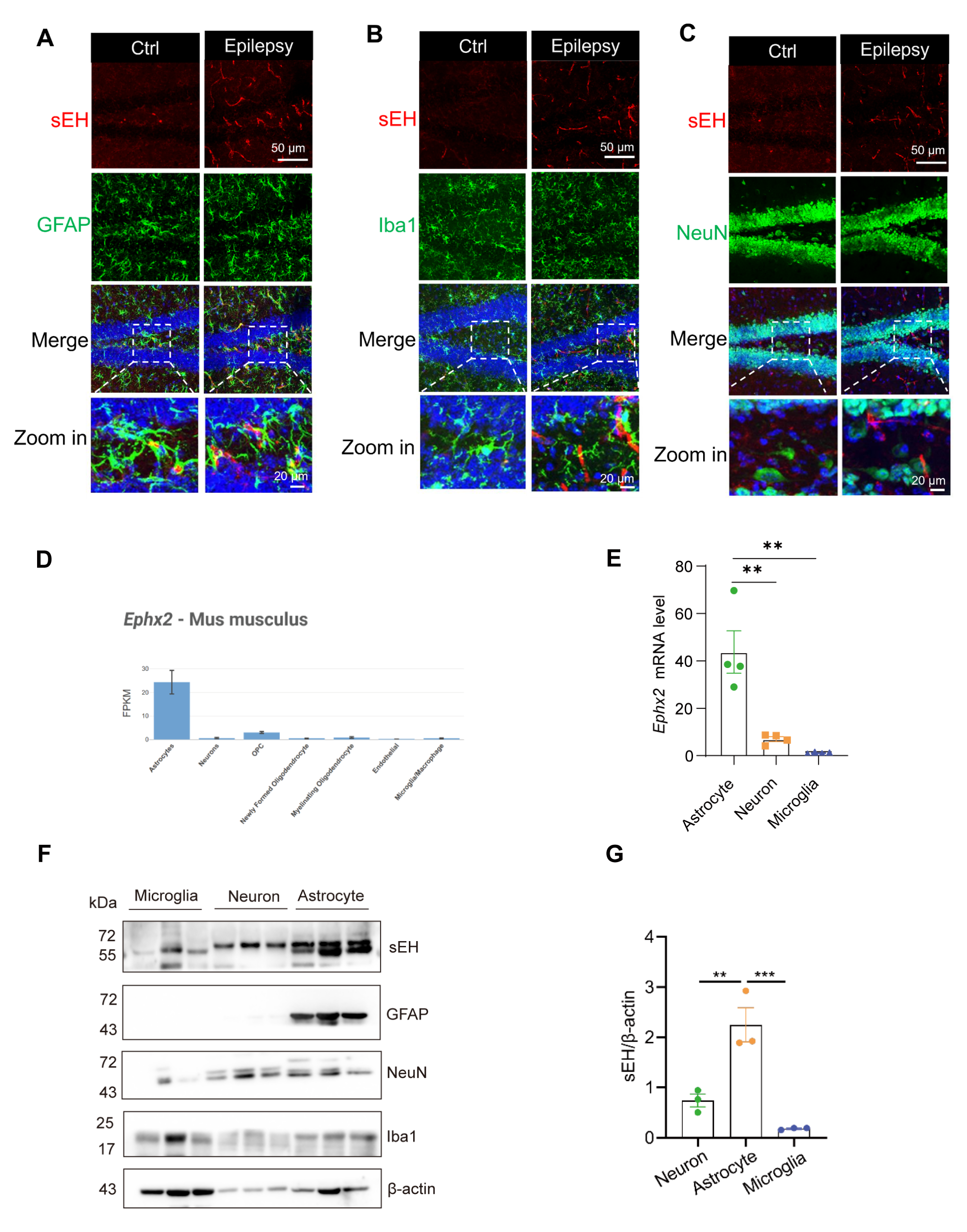

Supplement: Supplementary Figure 4 — Soluble epoxide hydrolase (sEH) is predominantly expressed in astrocytes in the CNS and is increased following epilepsy induction. (A) Representative immunofluorescent images of sEH localization in the control (Ctrl) or epilepsy (KA) brain slices labelled using GFAP (green) and sEH (red) antibodies. (B) Representative immunofluorescent images of sEH localization in the control (Ctrl) or epilepsy (KA) brain slices labelled using Iba1 (green) and sEH (red) antibodies. (C) Representative immunofluorescent images of sEH localization in the control (Ctrl) or epilepsy (KA) brain slices labelled using NeuN (green) and sEH (red) antibodies. (D) The transcriptional levels of the sEH coding gene mus-Ephx2 in different cell types in the brain from the brain-seq database. (E) Detection of the transcriptional levels of Ephx2 in primary cultured neurons, astrocytes, and microglia using quantitative real-time PCR. (F) Representative images of Western blotting for sEH in different cell types are shown. (G) Protein levels of sEH were quantified by densitometry and are presented as ratios to β-actin. Data are presented as mean ± standard error of the mean (SEM), n=3, One-way ANOVA, **p<0.01, ***p<0.001. [file Image4.tif]

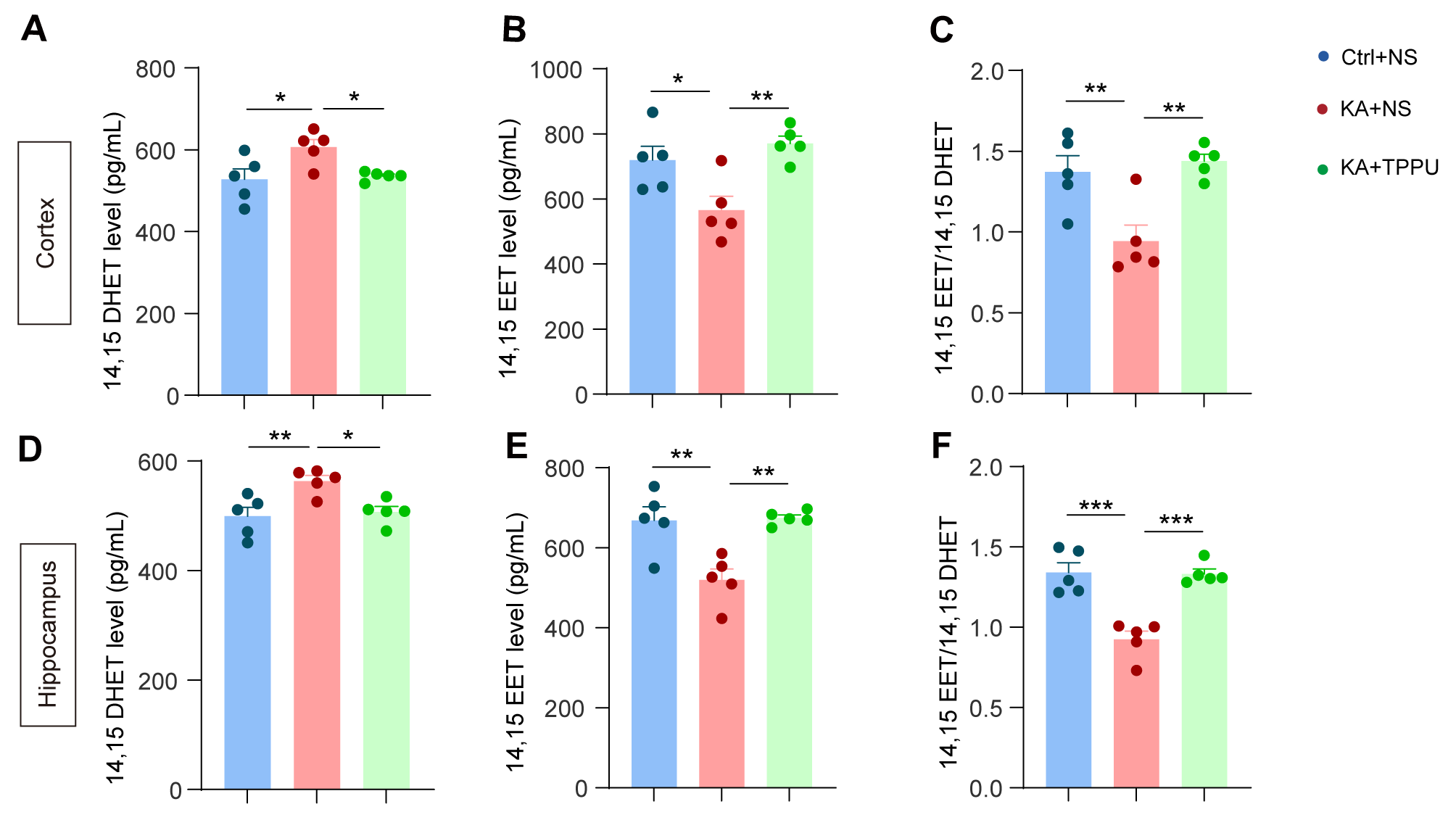

Supplement: Supplementary Figure 5 — TPPU inhibits the activity of sEH in chronic epileptic mice. (A) The levels of 14,15-DHET, a sEH catalytic product, in the cortex of mice from the control group (Ctrl+NS), epileptic group (KA+NS), and TPPU treatment group (KA+TPPU) were detected by ELISA. (B) The levels of 14,15-EET in the cortex of mice from the control group (Ctrl+NS), epileptic group (KA+NS), and TPPU treatment group (KA+TPPU) were detected by ELISA. (C) The ratio of 14,15-EET/14,15 DHET in the cortex of mice from the control group (Ctrl+NS), epileptic group (KA+NS), and TPPU treatment group (KA+TPPU). (D) The levels of 14,15-DHET, a sEH catalytic product, in the hippocampus of mice from the control group (Ctrl+NS), epileptic group (KA+NS), and TPPU treatment group (KA+TPPU) were detected by ELISA. (E) The levels of 14,15-EETin the hippocampus of mice from the control group (Ctrl+NS), epileptic group (KA+NS), and TPPU treatment group (KA+TPPU) were detected by ELISA. (F) The ratio of 14,15-EET/14,15 DHET in the hippocampus of mice from the control group (Ctrl+NS), epileptic group (KA+NS), and TPPU treatment group (KA+TPPU). Data are presented as mean ± standard error of the mean (SEM), n=5, One-way ANOVA, *p<0.05, **p<0.01. [file Image5.tif]

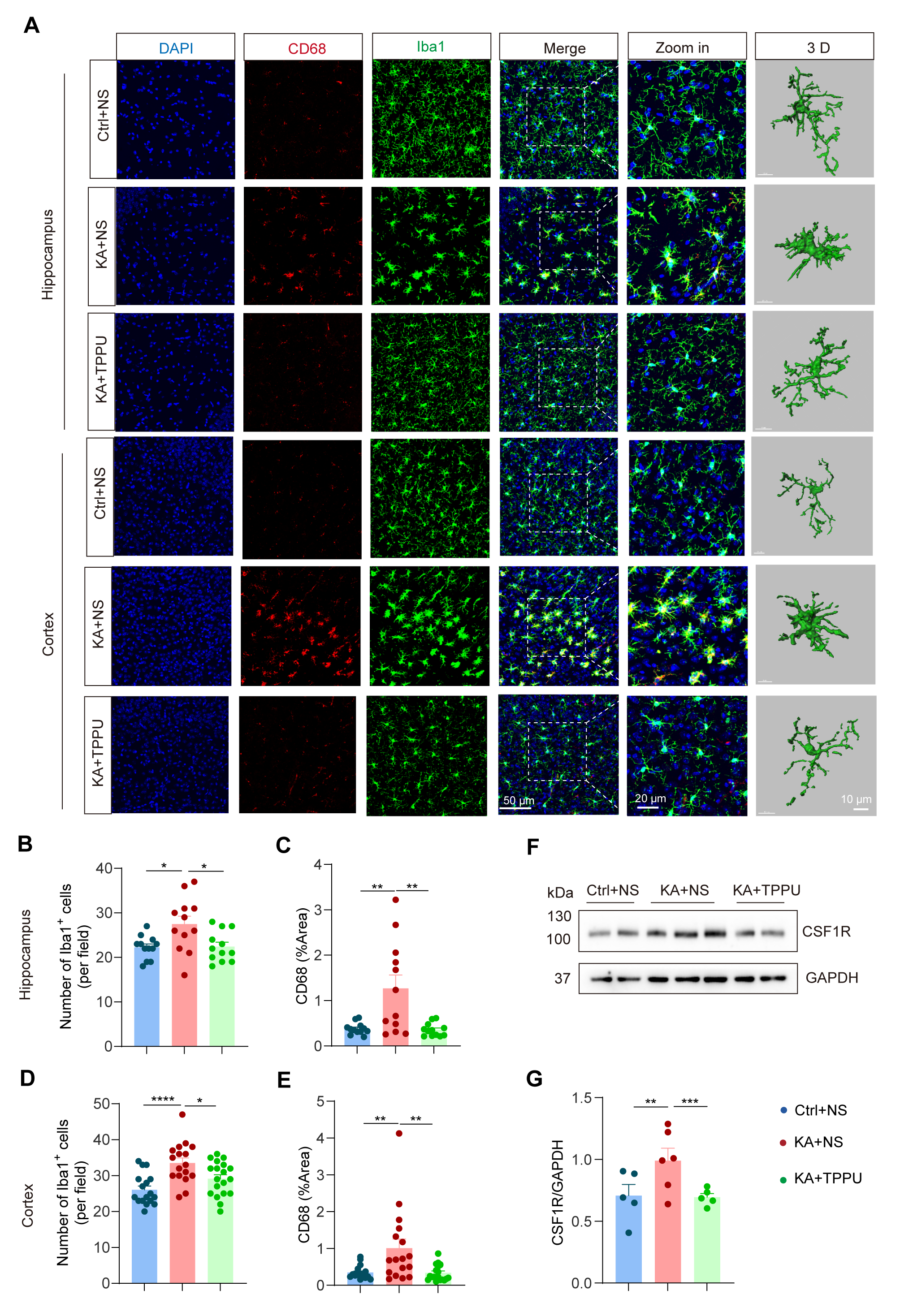

Supplement: Supplementary Figure 6 — TPPU inhibits microglial activation in chronic epileptic mice. (A) Representative images of hippocampal regions from the control (Ctrl) or epilepsy (epilepsy) brain mice treated with or without TPPU. Slices were labelled using Iba1 (green) and CD68 (red) antibodies. (B) The number of Iba1 (green) microglia per high-power field in the hippocampus of those mice. (C) The percentage of Iba1 (green) and CD68 (red) positive (GFAP+ sEH+) microglia relative to Iba1+ microglia per high-power field in the hippocampus of those mice. (D) The number of Iba1 (green) microglia per high-power field in the cortex of those mice. (E) The percentage of Iba1 (green) and CD68 (red) positive (GFAP+ sEH+) microglia relative to Iba1+ microglia per high-power field in the cortex of those mice. (F) Representative images of Western blot for CSF1R in the hippocampal tissue of Ctrl+NS, KA+NS, and KA+TPPU mice. (G) Protein levels of CSF1R were quantified by densitometry and are presented as ratios to GAPDH. Data are presented as mean ± standard error of the mean (SEM), n=4-6, One-way ANOVA, *p<0.05, **p<0.01, ***p<0.001, ****p<0.0001. [file Image6.tif]

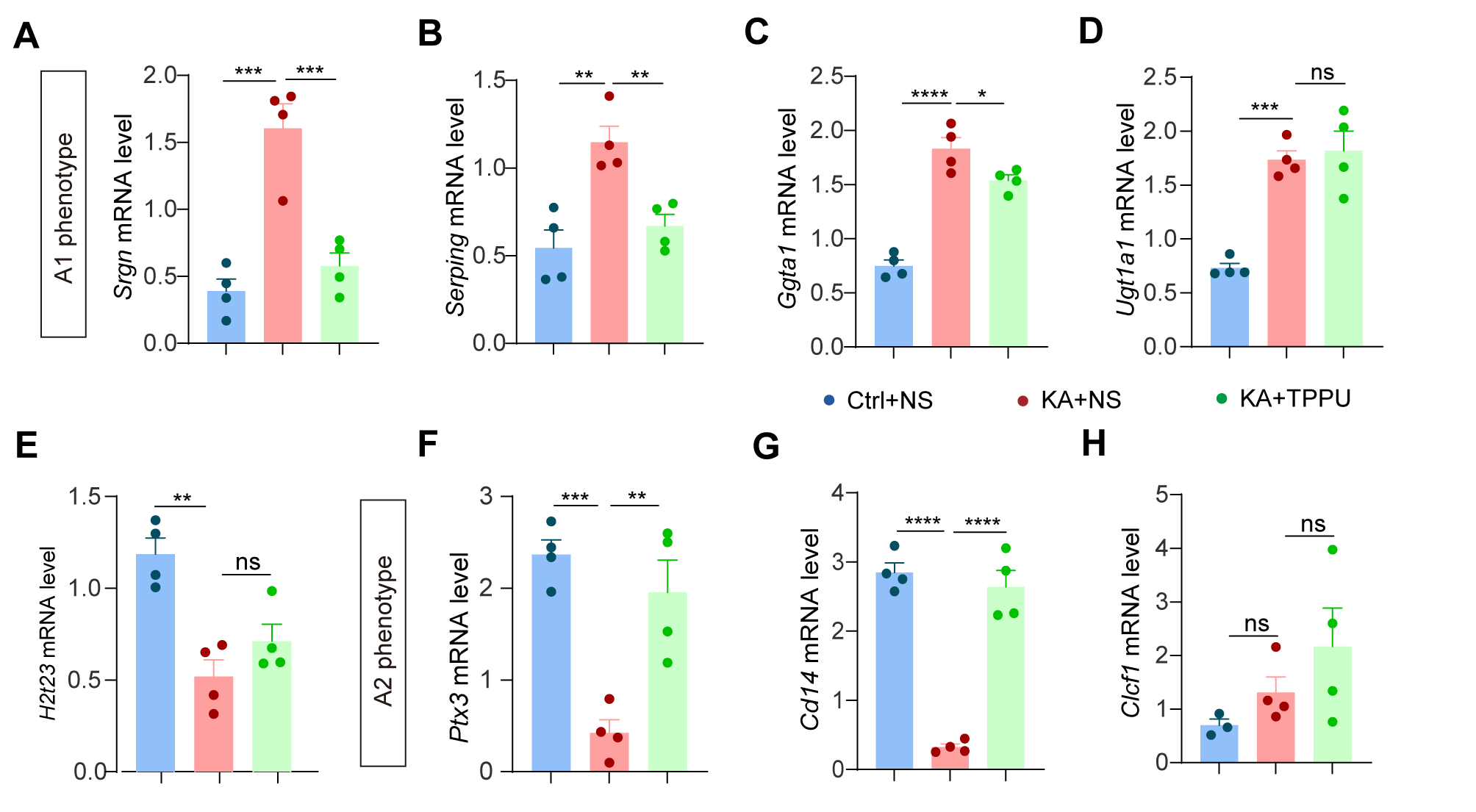

Supplement: Supplementary Figure 7 — TPPU inhibits the activation of reactive astrocytes. (A–E) Primary astrocytes were treated with KA (100 μM) and TPPU (100 μM) for 24 hours, and the transcriptional expression levels of A1 reactive astrocyte-related markers Srgn, Serping, Ugt1a1, Ggta1, and H2t23 were assessed using quantitative real-time PCR. (F–H) The transcriptional expression levels of A2 reactive astrocyte-related markers Ptx3, Cd14, and Clcf1 were measured by quantitative real-time PCR. Data are presented as mean ± standard error of the mean (SEM), n=3, One-way ANOVA, *p<0.05, **p<0.01, ***p<0.001, ****p<0.0001, ns: no statistical difference. [file Image7.tif]

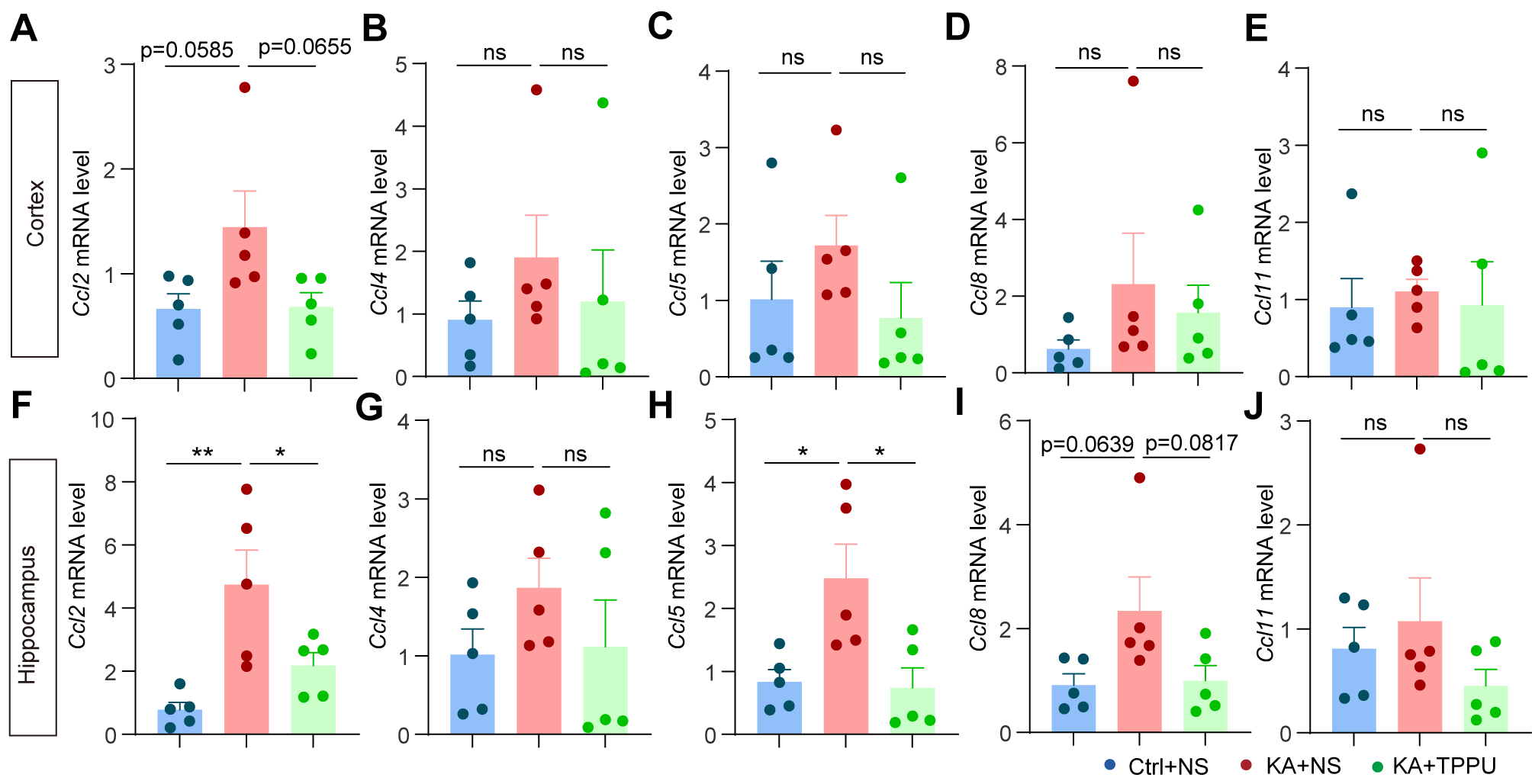

Supplement: Supplementary Figure 8 — TPPU inhibits neuroinflammation in chronic epileptic mice. (A–E) Detection of the transcriptional levels of cytokine Ccl2, Ccl4, Ccl5, Ccl8 and Ccl11 in the cortex of mice from control group (Ctrl+NS), epileptic group (KA+NS), and TPPU treatment group (KA+TPPU) using RT-PCR. (F–J) Detection of the transcriptional levels of cytokine Ccl2, Ccl4, Ccl5, Ccl8 and Ccl11 in the hippocampus of mice from control group (Ctrl+NS), epileptic group (KA+NS), and TPPU treatment group (KA+TPPU) using RT-PCR. N=5. One-way ANOVA, *p<0.05, **p<0.01, ns, not significant. [file Image8.tif]

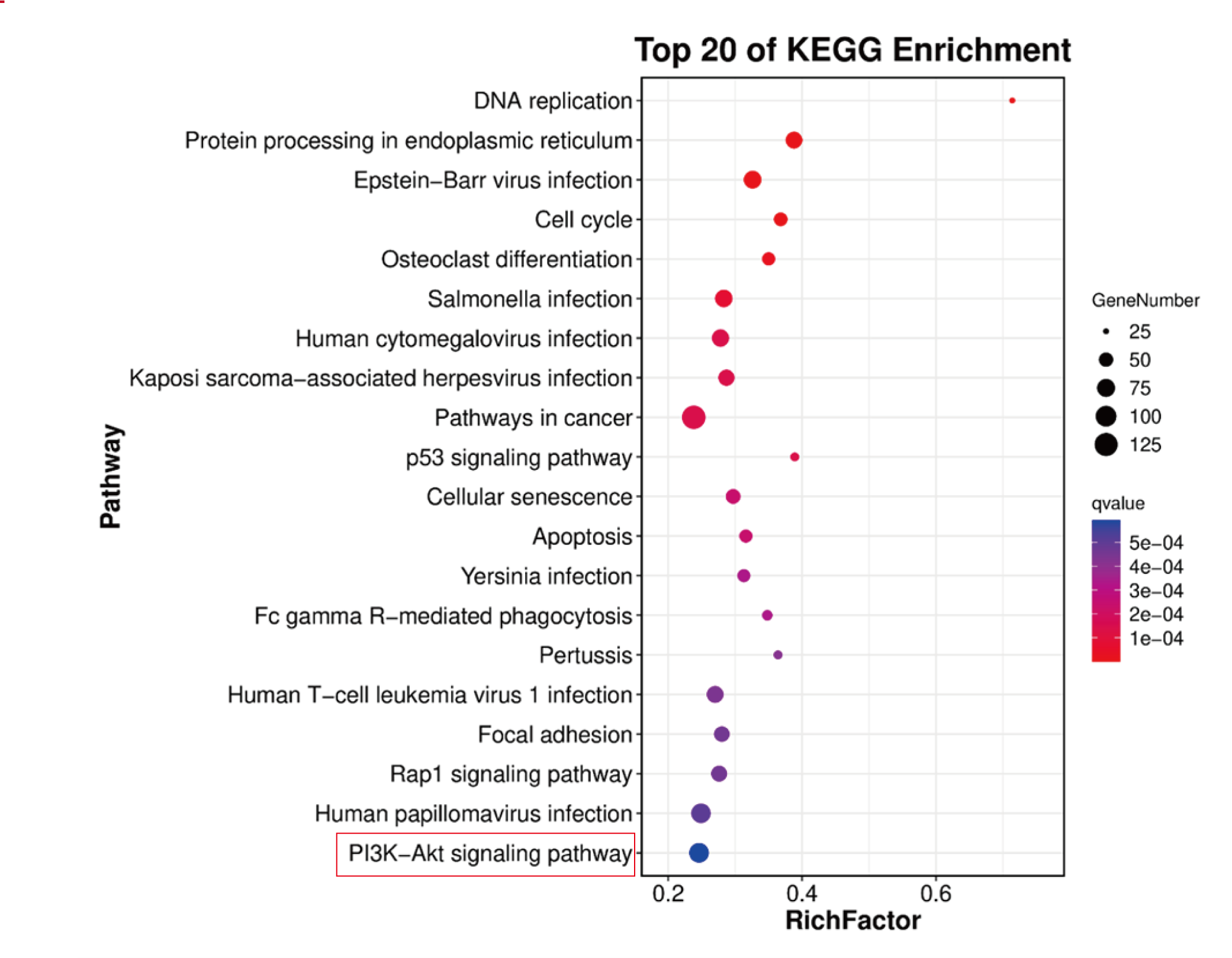

Supplement: Supplementary Figure 9 — KEGG pathway enrichment analysis on WGCNA turquoise module. PI3K-Akt signaling pathway was significantly enriched, red box highlighted. [file Image9.tif]
